# Supplementary material for: Intraurban socioeconomic inequalities in life expectancy: a population-based cross-sectional analysis in the city of Córdoba, Argentina (2015-2018)
Source: BMJ Open. Author manuscript; Available in PMC 2022 Oct 8. (PMC9442478; doi:10.1136/bmjopen-2022-061277)

Supplementary material

Modeling the mortality rate

We employ the Bayesian adaptation of the tool for projecting age patterns using linear splines method (TOPALS) which incorporates spatial smoothing in small areas.[19]

For age $a\ \{a = 0,1,2,\ldots,85\}$ and small area $i\ \{i = 1,2,\ldots,99\}$ with corresponding population n_{ia}, we assume that the number of deaths comes from a Poisson distribution with underlying rate λ_{ia}.

$$y_{ia}|\ \lambda_{ia} \sim Pois\ (n_{ia}\ \lambda_{ia})$$

We further assume that the vector of log mortality rates in small area i is,

$$log(\boldsymbol{\lambda}_{i.}) = log(\boldsymbol{\lambda}^{*}) + \boldsymbol{B}\ \boldsymbol{\beta}_{\boldsymbol{k}.},$$

Where $\boldsymbol{\lambda}_{i.}$ is the vector with elements λ_{ia} representing age-specific mortality rates in small areas, $\boldsymbol{\lambda}^{*}$ is the vector of standard mortality schedule (i.e., the smoothed city-level rates), $\boldsymbol{B}$ is a matrix of constants of size 86×7 in which each column is a linear B-spline basis function, and $\boldsymbol{\beta}_{\boldsymbol{k}.}$ is a vector of parameters with elements β_{ik} representing offsets to the standard schedule.

We define knots at ages $t_0,\ldots,t_6$ = (0, 1, 10, 20, 40, 70, 85). For ages a in {0, 1, 2, ..., 85} and columns k in {1,...,7} the basis functions in $\boldsymbol{B}$ are:

$$\frac{a - t_{k-1}}{t_k - t_{k-1}}\ if\ t_{k-1} \le a \le t_k;$$

$$\frac{t_{k+1} - a}{t_{k+1} - t_k}\ if\ t_k \le a \le t_{k+1};$$

$$0\ \ otherwise.$$

We further decompose the β_{ik} into the intercepts at each knot, β_{0k}; the spatial random effects, z_{ik}; and unstructured random effects, ϕ_{ik} that vary by knot age and area.

$$\beta_{ik} = \beta_{0k} + z_{ik} + \phi_{ik}\ .$$

We assign the unstructured, non-spatial random effect (ϕ_{ik}) an exchangeable zero-mean normal prior with the knot-specific variance $\sigma^2_{ns;\,k}$. The variance parameter in turn receives the uninformative inverse gamma hyper-prior with the shape and rate parameter of 1 and 0.01, respectively.

That is,

$$\phi_{ik} \sim Normal\ (0, \sigma^2_{ns;\,k})$$
$$\sigma^2_{ns;\,k} \sim Inverse\ Gamma\ (1, 0 \cdot 01)$$

For the intercept β_{0k}, we assign a vague normal prior with mean 0 and variance of 1,000. That is,

$$\beta_{0k} \sim Normal\ (0, 1000)$$

For the spatial random effect z_{ik}, we assign the intrinsic conditional autoregressive (ICAR) prior distribution[45] for each knots, k. We define areas i and j as neighbors if they share one or more common vertex between boundaries, commonly referred to as Queen's contiguity.

For any given knot and for each area i, the conditional expected value of z_{ik} is the mean of its neighboring areas, and the variance of z_{ik} is inversely proportional to the number of neighbors for that area, m_i.

If we drop the subscript for knot here, we can denote the CAR distribution as:

$$z_i|z_{-i}, \boldsymbol{W},\ \sigma_z^2 \sim Normal\ (\bar{z}_i, \frac{\sigma_z^2}{m_i}),$$

where

$$\bar{z}_i = \sum_{j,j\neq i} \frac{w_{i,j}\ z_j}{m_i}$$

and $W = [w_{i,j}]$ is 99×99 adjacency matrix with elements $w_{i,j} = 1$ if areas i and j are neighbors and 0 otherwise. We complete the prior specification by assigning a weakly informative inverse gamma prior (1, 0·14) for the variance of CAR random effects.

Rodríguez López S, *et al. BMJ Open* 2022; 12:e061277. doi: 10.1136/bmjopen-2022-061277

Figure S1. Log mortality rates due to all causes for women and men. Córdoba, 2015-2018.

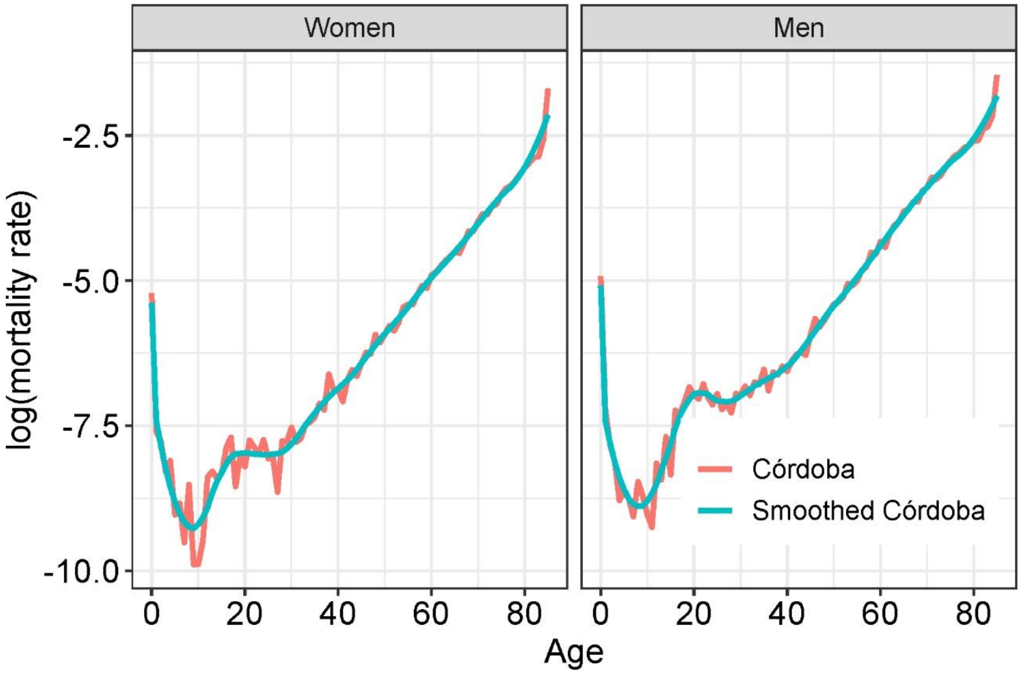

Footnote: because log mortality rates of the standard mortality schedule (labelled Córdoba) were noisy (i.e., contained unexplained variance), we fit a LOESS regression and the resulting LOESS-smoothed curve (labelled Smoothed Córdoba) served as the standard mortality schedule in the analysis.

Figure S2. Life expectancy at birth (95% Credible Interval) among women and men in small areas. Córdoba, 2015-2018.

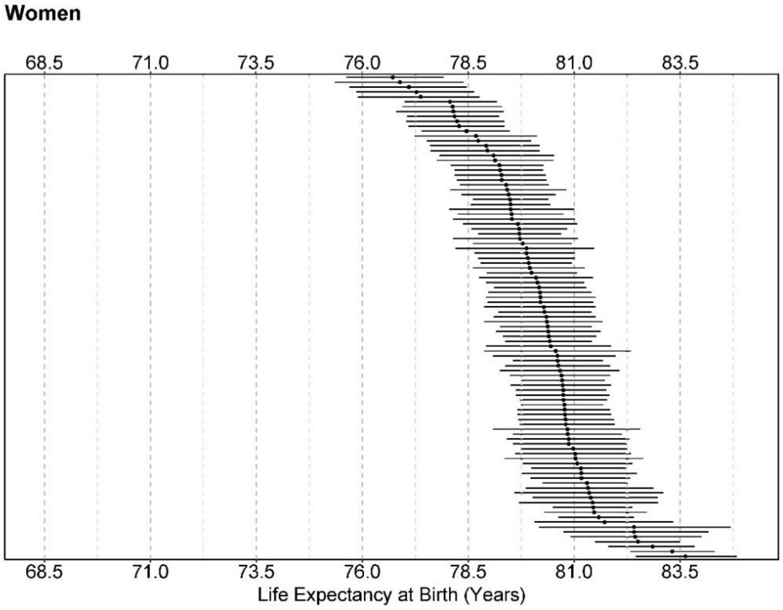

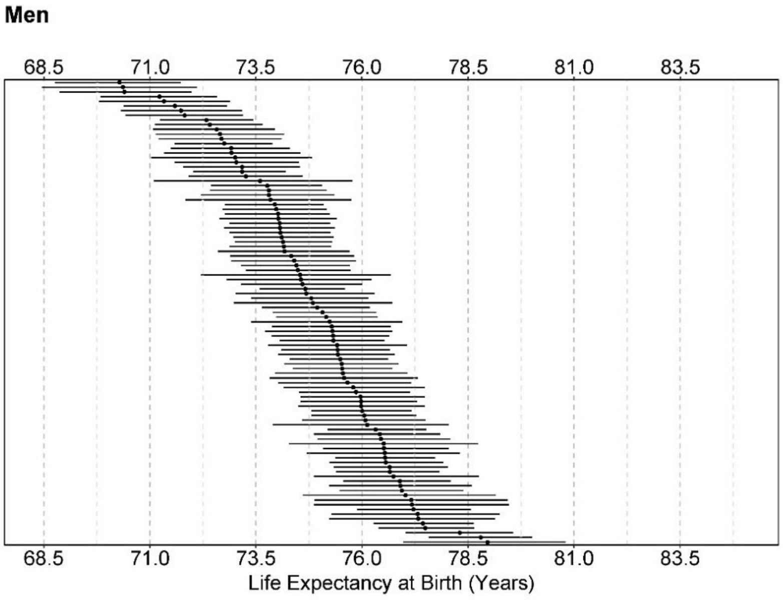

Footnote: small areas are represented in the vertical axis.

Figure S3. Spatial distribution of life expectancy at birth, 20, 40 and 60 years in women and men in the 99 small areas of city of Córdoba, 2015-2018.

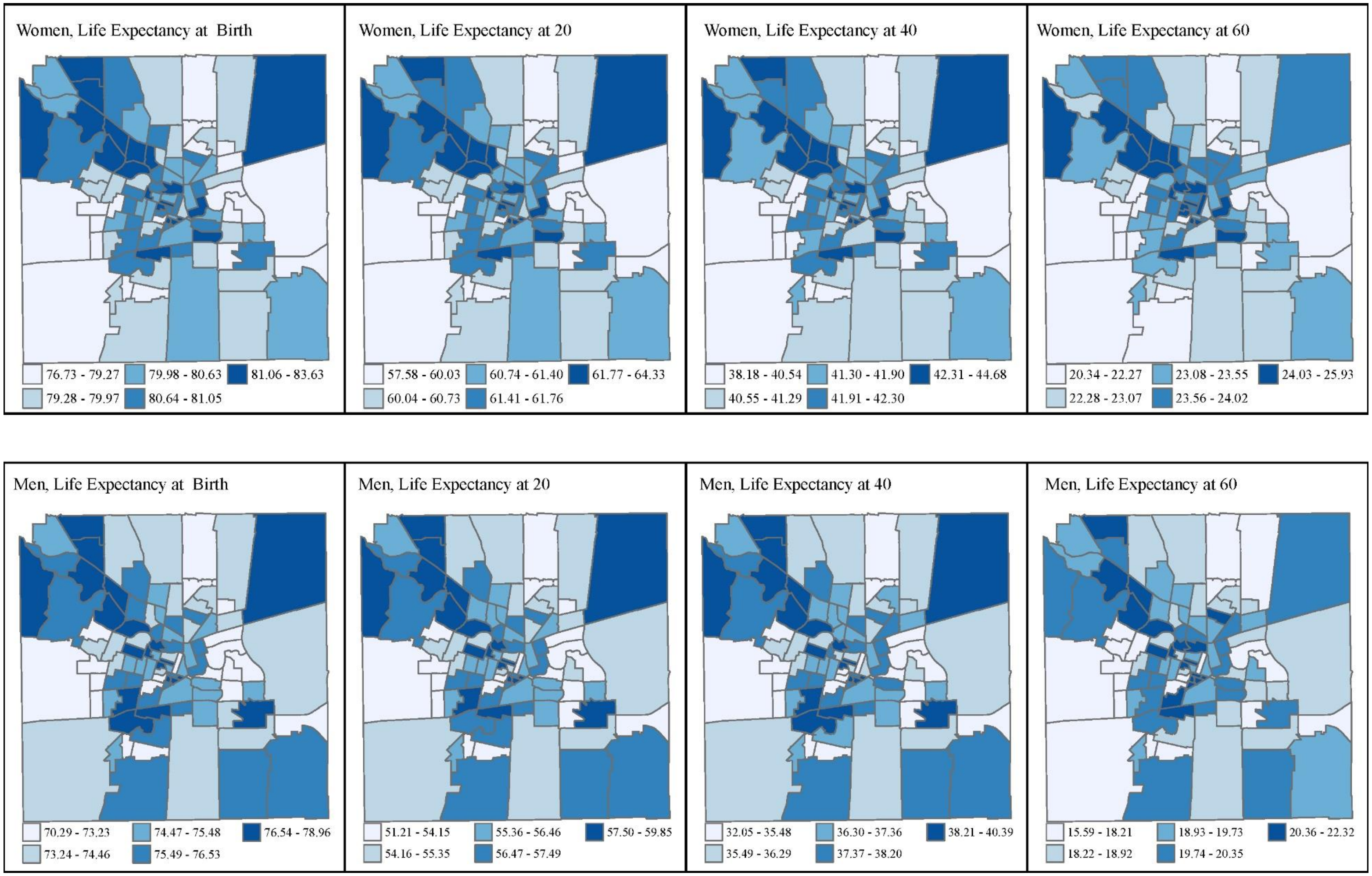

Supplement: Data supplement [file EMS154770-supplement-Data_supplement.pdf]
