# Supplementary material for: Intraurban socioeconomic inequalities in life expectancy: a population-based cross-sectional analysis in the city of Córdoba, Argentina (2015–2018)
Source: BMJ Open. 2022 Sep 2;12(9):e061277. doi: 10.1136/bmjopen-2022-061277 (PMC9442478; doi:10.1136/bmjopen-2022-061277)
Supplement: Supplementary file 1 [file bmjopen-2022-061277.draft_revisions.pdf]

BMJ Open is committed to open peer review. As part of this commitment we make the peer review history of every article we publish publicly available.

When an article is published we post the peer reviewers' comments and the authors' responses online. We also post the versions of the paper that were used during peer review. These are the versions that the peer review comments apply to.

The versions of the paper that follow are the versions that were submitted during the peer review process. They are not the versions of record or the final published versions. They should not be cited or distributed as the published version of this manuscript.

BMJ Open is an open access journal and the full, final, typeset and author-corrected version of record of the manuscript is available on our site with no access controls, subscription charges or pay-per-view fees (http://bmjopen.bmj.com).

If you have any questions on BMJ Open's open peer review process please email info.bmjopen@bmj.com

BMJ Open

Life expectancy in small areas of the city of Córdoba, Argentina (2015-2018)

Journal:	*BMJ Open*
Manuscript ID	bmjopen-2022-061277
Article Type:	Original research
Date Submitted by the Author:	21-Jan-2022

Complete List of Authors:	Rodríguez López, Santiago ; Consejo Nacional de Investigaciones Cientificas y Tecnicas, Centro de Investigaciones y Estudios sobre Cultura y Sociedad; UNC FCEFyN, Cátedra de Antropología<br>Tumas, Natalia; Consejo Nacional de Investigaciones Cientificas y Tecnicas, Centro de Investigaciones y Estudios sobre Cultura y Sociedad (CIECS, CONICET y UNC); Pompeu Fabra University, Ciències Polítiques i Socials<br>Bilal, Usama; Drexel University Dornsife School of Public Health, Urban Health Collaborative; Drexel University Dornsife School of Public Health, Epidemiology and Biostatistics<br>Moore, Kari; Drexel University School of Public Health, Urban Health Collaborative<br>Acharya, Binod; Drexel University Dornsife School of Public Health, Urban Health Collaborative<br>Quick, Harrison; Drexel University Dornsife School of Public Health, Urban Health Collaborative; Drexel University Dornsife School of Public Health, Epidemiology and Biostatistics<br>Quistberg, Alex; Drexel University Dornsife School of Public Health, Urban Health Collaborative<br>Acevedo, Gabriel; UNC FCM, Cátedra de Medicina Preventiva y Social<br>Diez Roux, Ana V.; Drexel University Dornsife School of Public Health, Urban Health Collaborative; Drexel University Dornsife School of Public Health, Epidemiology and Biostatistics

Keywords:	PUBLIC HEALTH, SOCIAL MEDICINE, EPIDEMIOLOGY

SCHOLARONE™ Manuscripts

I, the Submitting Author has the right to grant and does grant on behalf of all authors of the Work (as defined in the below author licence), an exclusive licence and/or a non-exclusive licence for contributions from authors who are: i) UK Crown employees; ii) where BMJ has agreed a CC-BY licence shall apply, and/or iii) in accordance with the terms applicable for US Federal Government officers or employees acting as part of their official duties; on a worldwide, perpetual, irrevocable, royalty-free basis to BMJ Publishing Group Ltd ("BMJ") its licensees and where the relevant Journal is co-owned by BMJ to the co-owners of the Journal, to publish the Work in this journal and any other BMJ products and to exploit all rights, as set out in our licence.

The Submitting Author accepts and understands that any supply made under these terms is made by BMJ to the Submitting Author unless you are acting as an employee on behalf of your employer or a postgraduate student of an affiliated institution which is paying any applicable article publishing charge ("APC") for Open Access articles. Where the Submitting Author wishes to make the Work available on an Open Access basis (and intends to pay the relevant APC), the terms of reuse of such Open Access shall be governed by a Creative Commons licence – details of these licences and which Creative Commons licence will apply to this Work are set out in our licence referred to above.

Other than as permitted in any relevant BMJ Author's Self Archiving Policies, I confirm this Work has not been accepted for publication elsewhere, is not being considered for publication elsewhere and does not duplicate material already published. I confirm all authors consent to publication of this Work and authorise the granting of this licence.

For peer review only

Life expectancy in small-areas of the city of Córdoba, Argentina (2015-2018)

Santiago Rodríguez López*[1,2]; Natalia Tumas[1,3]; Usama Bilal[4,5]; Kari Moore[4]; Binod Acharya[4]; Harrison Quick[4,5]; Alex D. Quistberg[4]; Gabriel E. Acevedo[6]; Ana V. Diez Roux[4,5]

[1] Centro de Investigaciones y Estudios sobre Cultura y Sociedad, Consejo Nacional de Investigaciones Científicas y Técnicas y Universidad Nacional de Córdoba (CIECS, CONICET y UNC), Argentina.

[2] Cátedra de Antropología, Facultad de Ciencias Exactas, Físicas y Naturales, Universidad Nacional de Córdoba, Argentina.

[3] Departament de Ciències Polítiques i Socials, Universitat Pompeu Fabra, Barcelona, Spain.

[4] Urban Health Collaborative, Dornsife School of Public Health, Drexel University, Philadelphia, USA.

[5] Department of Epidemiology and Biostatistics, Dornsife School of Public Health, Drexel University, Philadelphia, USA.

[6] Cátedra de Medicina Preventiva y Social, Facultad de Ciencias Médicas, Universidad Nacional de Córdoba, Argentina.

***Corresponding author:** Santiago Rodríguez López. Centro de Investigaciones y Estudios sobre Cultura y Sociedad (CIECS, CONICET y UNC), Av. Valparaíso s/n, Ciudad Universitaria, Córdoba, Argentina. C.P: 5000. +54 351 535 3769. e-mail: santiago.rodriguez@conicet.gov.ar

Keywords: life expectancy; neighborhoods; Argentina; TOPALS

Word count: 3,775

For peer review only

Abstract

Objectives: To evaluate the variability in life expectancy at birth in small areas, describe the spatial patterning of life expectancy at birth, and examine the association between small-area socioeconomic characteristics and life expectancy at birth.
Design: Cross-sectional, using data compiled by the SALURBAL project (Urban Health in Latin America) on: (1) death registries (2015-2018), including (2) population projections by age, sex, and municipality (2015-2018); and (3) population and socioeconomic characteristics data from the 2010 national population census.
Participants/setting: 40,898 records for 2015-2018 in 99 small areas of the city of Córdoba (Argentina).
Primary outcome: Life expectancy at birth. We analyzed variability in life expectancy at birth (P90-P10 gap) across small areas and evaluated the association with small-area socioeconomic characteristics using linear regression.
Results: The median life expectancy at birth was 80.3 years in women (P90-P10 gap=3.2 years) and 75.1 years in men (P90-P10 gap=4.6 years). We found higher life expectancies in the core part of the city and a spatial pattern by which life expectancy was highest in the core and the northwest, especially among women. We found positive associations between better small-area socioeconomic characteristics and life expectancy, especially among men: mean differences in life expectancy in years per SD higher value of area characteristics in men (women) were 0.97 (0.83), 1.04 (0.80), and 0.60 (0.55) for % adults with high school education or above, % persons aged 15-17 attending school and % with water inside the dwelling, respectively. Lower values of % overcrowded households and unemployment rate were associated with higher life expectancy: mean differences per SD lower values were 0.86 and 0.89 in men and 0.75 and 0.76 years in women respectively.
Conclusion: Our study provided the first characterization of the heterogeneity in life expectancy in small areas of Córdoba, suggesting a social patterning of longevity.

Strenghs and limitations

- The Bayesian approach draws strength and smooths estimates from surrounding small areas and from the overall structure of the mortality schedule.
- Univariate models with multiple imputation type techniques acknowledge uncertainty around the estimates of life expectancy.
- There is a relatively high proportion of records with low georeferencing accuracy, i.e., that were georeferenced to the street level.
- Applying spatial and age smoothing to address the small-area estimation of mortality rates, may have reduced inequalities.
- We assume that the social characteristics of small areas, the population age and sex structure were relatively stable acroos the years examined.

For peer review only

Introduction

The unequal social context that surrounds daily life of more disadvantaged groups is linked to worse health outcomes.[1] Health inequalities tend to be stronger in urban areas because they include areas of concentrated deprivation and poverty,[2,3] especially in large cities where spatial and social inequalities are combined.[4] In Latin America, one of the most unequal regions in the world with more than half of the world's most unequal cities,[5] there are large spatial differences in life expectancy, and these are strongly linked to social factors.[6–8]

Argentina, one of the most urbanized countries of Latin America, is an upper-middle-income country with large social and health disparities.[9] However, evidence on intraurban health variations across small areas is still scarce. Prior research showed that higher income inequality at the provincial level was associated with lower life expectancy for both men and women.[10] Another study described significant associations between social inequity and premature death rate in departments of Argentina.[11] Diez Roux et al.[4] described substantial intraurban variation in the risk of death in the core area of the large urban agglomeration of Buenos Aires, finding similar patterns to another study examining life expectancy across the entire metropolitan area.[8] These studies are primarily focused on the city of Buenos Aires, but no studies of which we are aware have so far addressed variability and the social patterning of life expectancy at birth in Córdoba, the second most populated city in the country.

Informed urban policies based on evidence about the heterogeneity in social and health indicators within cities are important to reduce inequities in health. However, many social and economic drivers of health disparities across small areas remain underrecognized.[12] As is the case for other large cities, generating local evidence to fill this knowledge gap on existing social inequities in life expectancy at birth in the city of Córdoba is relevant to inform policies to improve health conditions.[13] To fill this gap in knowledge we: 1) evaluated the variability in life expectancy at birth in small areas of the city of Córdoba for the 2015-2018 period; 2) described the spatial patterning of life expectancy at birth; and 3) examined the association between small area socioeconomic characteristics and life expectancy at birth. We hypothesized that (1) there is considerable variability of life expectancy at birth across small areas of Córdoba; (2) there is lower life expectancy in the periphery of the city of Córdoba as compared to core areas; and (3) small areas with better socioeconomic characteristics have higher life expectancy at birth.

Methods

Study setting

We used data compiled by the SALURBAL project (Urban Health in Latin America; 'Salud Urbana en América Latina') for the city of Córdoba during the years 2015-2018, which has compiled and harmonized data on health, social, and built environment indicators for a large number of cities in different Latin American countries.[14] The city of Córdoba (Argentina), capital and main city of Córdoba province, is a highly urbanized area of 576 km^2 and around 1.3 million residents. We defined small-areas as the 99 *fracciones censales* (spatially delimited units used for census data collection). These small areas had a median population (5th-95th percentile) of 13,370 (5,837-22,031) individuals in 2010 (median annual average for the 2015-2018 period: 14,389 (6,305-23,567)).

For peer review only - http://bmjopen.bmj.com/site/about/guidelines.xhtml

Data Sources

We used data from (1) death registries geocoded to small areas during the years 2015-2018, (2) population projections by age, sex, and municipality for 2015-2018, and (3) population and socioeconomic characteristics by small area from the 2010 national population census of Argentina.[15]

Mortality Data

We used all mortality records for the city of Cordoba from 2015 to 2018 (n= 42,115). We geocoded these records to small areas, and excluded records georeferenced to areas outside of the administrative boundaries of Córdoba (n=1,084) and to non-residential points of interest (n=133). Of the remaining 40,898 geocoded death records, n=4,075 (~10%) had low georeferencing accuracy (i.e., matched to streetname but not building number) but could still be assigned to a small area using the center point of the street or area they were georeferenced to and were included in the study. The analysis comprised a total of 40,898 deaths for the 2015-2018 period in 99 small areas.

Population Data

We obtained population denominators by sex, single years of age (0 to 110+ years), and small areas. First, we we used 2010 census data (available at the small area level) to calculate the proportion of people in the city of Cordoba by age, sex and small area. Second, we obtained population projections for 2015-2018 by 5-year age groups (0 to 85+ years) and sex for the whole municipality of Cordoba, and graduated this data into single ages using a penalized composite link model (PCLM).[16] We then obtained small-area population by single age and sex for 2015-2018 by applying the 2010 census proportions to the graduated city-level level population (2015-2018), obtaining small-area population by single year age and sex.

Socioeconomic characteristics of small areas

We used the following variables to proxy socioeconomic characteristics of small areas: % of the population aged 15 to 17 years attending school, % of the population aged 25 years or above with completed secondary education or above, % of households with piped water access inside the dwelling, % households with overcrowding (defined as more than three people per room), and % unemployment among age 15 years and above. Area-level education has been associated with variations in intra-urban mortality in Argentina,[4,8] and water access, sanitation, and less overcrowding[7] have been also associated with higher life expectancy in Latin American cities. In addition, and in order to capture broader features of the social environment, we created a composite z-score index based on the variables listed above. Although a multidimensional index only captures some aspects of the urban infrastructure and resources,[7] it is likely a better proxy of the social environment than a single indicator. Combined indices including education indicators have been frequently used in health disparities research.[17,18] We standardized each of these variables so that they have mean 0 and standard deviation 1. Unemployment and overcrowding were reversed (i.e. multiplied by -1). The average of these standardized scores was defined as the composite z-score representing the socioeconomic characteristics of the small areas.

Statistical analysis

To estimate life expectancy, we need an estimate of age-, sex-, and small-area specific mortality rates. We estimated these through a Bayesian adaptation of the tool for projecting age patterns using linear

For peer review only - http://bmjopen.bmj.com/site/about/guidelines.xhtml

splines method (TOPALS), incorporating spatial smoothing between small areas.[19] Projecting age patterns using a linear spline method (TOPALS regression) requires the use of a standard mortality schedule.[19] The core idea is that the schedule of log mortality rates to be estimated is the sum of the standard schedule and a linear spline function. Following the original TOPALS method,[19] we used the city-level Córdoba mortality schedule as the standard schedule. Other research has shown that the choice of this mortality schedule does not fundamentally alter results.[20] Because the log mortality rates of the standard mortality schedule were noisy (see Supplementary material, Figure S1), we fitted a LOESS regression and the resulting smoothed rates were used as the standard schedule for mortality. The models were run using the WinBUGS[21] by calling the software with R2WinBUGS package[22] in R for 50,000 iterations for each of the two chains. For each chain, the first 40,000 samples were discarded as burn-ins and the remaining samples were thinned by a factor of 10 to reduce autocorrelation of the samples. We fit the models for women and men separately. Following the fitting of this model, we sampled 2,000 sets of age-, sex-, and small-area specific mortality rates from the posterior distributions resulting from the model. See the Supplementary material for further details on how mortality rates were modeled.

To calculate life expectancy at birth, we inputted age- and sex-specific mortality rates into single-age life tables using the DemoTools package in R.[23] Period life expectancy at birth is a simple indicator to present differences in mortality across and within populations,[24] interpreted as the number of years someone born today is expected to live if current age-specific mortality patterns hold constant in the future. These life tables were calculated for each iteration of posterior samples, resulting in 1,000 sex- and small-area specific life expectancy at birth estimates. For descriptive purposes, we report a point estimate (median) and 95% credible intervals (2.5th and 97.5th percentiles). We also extracted life expectancy at ages 20, 40, and 60 from life tables.

To estimate the amount of variability in life expectancy at birth for each sex (hypothesis 1), we calculated the difference between 90th and the 10th percentile of the distribution of life expectancy at birth (P90-P10 gap) across small areas. To analyze the geographical patterning of life expectancy at birth (hypothesis 2), we mapped life expectancy at birth for men and women by small area using ArcGIS. Finally, to examine the association between life expectancy at birth and socioeconomic characteristics of small areas (hypothesis 3), we fitted univariate linear regressions of life expectancy on each standardized socioeconomic variable and the composite index, all in separate models. To acknowledge uncertainty around the estimates of life expectancy, these linear regressions were repeated 2,000 times, one per life expectancy estimate resulting from each posterior sample. We used Rubin's formula to pool coefficients to obtain a single regression coefficient and associated standard errors. Linear regression models were run using PROC REG and PROC MIANALYZE in SAS software.

Patient and Public Involvement

SALURBAL has launched a series of Knowledge-to-Policy Fora to present preliminary results and engage urban health policy actors from across Latin America in dialogue on urban health research and policy priorities in the region. For more information see https://drexel.edu/lac/events-workshops/knowledge-policy-forum/. The project also holds and participates in additional periodic workshops to engage stakeholders in activities designed to disseminate research findings and engage stakeholders in systems thinking around urban policies.

For peer review only - http://bmjopen.bmj.com/site/about/guidelines.xhtml

Results

A median of 222 and 199 deaths occurred during the study period per small area among women and men, respectively (Table 1). A median of 85.3% of adolescents among 15-17 years attended school, 58.4% of people had minimum high school education, and 7.0% were unemployed (Table 1). Moreover, 97.6% of dwellings had inside water connections and 2.1% of households experienced overcrowding. Compared to men, there was a lower proportion of women aged 15 or younger (22% women vs. 25.1% men) and a higher proportion of women aged 65 or older (13.3% vs. 8.9%) (Table 1). Median life expectancy at birth was 80.3 years for women with variability in life expectancy (P90-P10 gap) of 3.2 years (P90=81.5 years and P10=78.3 years). Among men, there was lower life expectancy at birth (75.1 years), with a gap (P90-P10) of 4.6 years between small areas (P90=77.1 years, and P10=72.5 years). For the full distribution of life expectancy at birth and uncertainty around the estimates see Figure S2 (Supplementary material).

Table 1. Number of deaths, life expectancy among women and men, and selected sociodemographic characteristics for small areas (n=99). Córdoba, 2015-2018.

	Median (10th–90th percentile)	
	Women	Men
Number of deaths	222 (90, 340)	199 (81, 309)
Estimated population, annual average [a]	7,326 (3,818, 11,157)	6,874 (3,214, 10,534)
Life expectancy at birth, years [a]	80.3 (78.3, 81.5)	75.1 (72.5, 77.1)
Life expectancy at age 20, years [a]	61.0 (59.1, 62.2)	56.2 (53.6, 57.9)
Life expectancy at age 40, years [a]	41.5 (39.8, 42.6)	36.9 (34.7, 38.6)
Life expectancy at age 60, years [a]	23.3 (21.6, 24.3)	19.2 (17.2, 21.0)
Population aged 15 or younger, % [a]	22.0 (8.7, 30.3)	25.1 (10.6, 32.2)
Population aged 65 or older, % [a]	13.3 (7.1, 19.5)	8.9 (5.3, 12.5)
Water inside dwelling, % [b]	97.6 (89.8, 99.4)	
Households with overcrowding, % [b]	2.1 (0.4, 7.1)	
School attendance among 15-17 years old, % [b]	85.3 (76.2, 91.9)	
Adults with high school education or above, % [b]	58.4 (30.1, 88.7)	
Unemployment, % [b]	7.0 (4.6, 9.7)	

Footnote: [a] Data from 2015-2018; [b] Data from 2010 census; Overcrowding: proportion of households with more than three people per room; Unemployment: proportion among the population aged 15 years or more in the labor force.

Figure 1 shows the distribution of life expectancy in small areas within the city. There were higher life expectancies in the core and northwestern parts of the city for both men and women, along with the southwestern part of the city for men (Figure 1). The distribution of life expectancy at ages 20, 40, and 60 years, showed similar patterns to those for life expectancy at birth in women and men (see Figure S3, Supplementary material).

[Figure 1]

Overall, there were positive associations between life expectancy at birth and better socioeconomic characteristics of small areas (Figure 2, Table 2), especially among men. In men, 1-SD higher value of high school education and school attendance was associated with higher life expectancy (mean differences 0.97 and 1.04 years, respectively in men and 0.83 and 0.80 years respectively in women

For peer review only - http://bmjopen.bmj.com/site/about/guidelines.xhtml

(Table 2). Similarly, 1-SD higher value of households with water inside the dwelling was associated with higher life expectancy by 0.6 years in men, and 0.55 years in women, while a 1-SD higher value of households with overcrowding and % unemployment was associated with lower life expectancy at birth by 0.86 and 0.89 years, respectively, and 0.75 and 0.76 years for women (Table 2). The composite z-score was also strongly associated with life expectancy, as a 1-SD higher score was associated with 1.07 and 0.91 years higher life expectancy in men and women, respectively.

Table 2. Mean differences in life expectancy (in years) associated with a 1-SD higher value of small area characteristics among women and men, Córdoba city, 2015-2018.

Variable/model	1 SD corresponds to	Mean difference in life expectancy (95% CI)	
		Women	Men
% with High school education or above	20.92%	0.83 (0.53, 1.13)	0.97 (0.57, 1.38)
% School attendance among adolescents (15-17 years)	6.38%	0.80 (0.51, 1.10)	1.04 (0.65, 1.42)
% households with water inside dwelling	4.59%	0.55 (0.24, 0.87)	0.60 (0.18, 1.01)
% households with overcrowding	2.85 %	- 0.75 (-1.05, -0.45)	- 0.86 (-1.26, -0.46)
% Unemployment	1.96%	- 0.76 (-1.06, -0.47)	- 0.89 (-1.29, -0.50)
Composite z-score	1 SD	0.91 (0.58, 1.24)	1.07 (0.64, 1.51)

Footnote: Beta coefficients represented the difference in life expectancy at birth (in years) associated with 1 SD higher value of the variable. The models were run in univariate fashion, one variable at a time. Socioeconomic data for small areas came from the 2010 census; Overcrowding: proportion of households with more than three people per room; Unemployment: proportion among the population 15 years or more in the labor force.

[Figure 2]

Discussion

Our study evaluated the variability in life expectancy at birth, described the spatial patterning of life expectancy and examined the association between life expectancy and socioeconomic characteristics of small areas in the city of Córdoba (Argentina) during the 2015-2018 period. We found a higher life expectancy for women (5.2 years higher than for men), that life expectancy varied within the city of Cordoba, with a P90-P10 gap of 4.6 years for men and 3.8 years for women, and that life expectancy was higher in the central and northwestern parts of the city, along with the southwestern part in the case of men. Lastly, we found that a series of small-area socioeconomic characteristics were highly predictive of life expectancy.

Analyses of spatial variability provide critical information for understanding place effects on health.[25] We found considerable variability (P90-P10 gap) in life expectancy at birth across small areas of Córdoba, with this gap being larger in men (4.6 years) than in women (3.8 years). Research describing variations in life expectancy and mortality across small areas within a single city in

For peer review only - http://bmjopen.bmj.com/site/about/guidelines.xhtml

Argentina has been mainly focused in the city of Buenos Aires.[4,26] In another study we also found that variability in rates of non-communicable disease risk factors was higher across neighborhoods within cities than across cities,[27] suggesting large heterogeneities among the leading causes of death in Argentina. Aligned with this, a study in the US showed that more than three-fourths of the total variation in life expectancy was attributable to census tract units, suggesting that population inequalities in longevity are primarily a local phenomenon.[28]

Other analyses of life expectancy gaps within cities revealed wide gaps. Within Latin American cities[8], intraurban life expectancy ranged in women from a low of 3.0 years in San José to a high of 17.7 years in Santiago, and in men, from a low of 4.0 years in Belo Horizonte to a high of 15.0 years in Panama city. A study in Rio de Janeiro[29] showed that life expectancy at birth of men living in the richest parts of the city was 12.8 years longer than that of men living in deprived areas. In Rome[30], differences in life expectancy among districts in the historical center and districts on the outskirts of the city were up to about 2.7 years for men and 2.1 years for women. In Scotland[31], there was also a big gap in life expectancy between the least and most deprived areas of about 13 years for men and around 10 years for women. Using a similar unit of analysis to ours, other studies described variations across small-area life expectancy: Boing et al.[28] found a 13.1-year gap in census tract life expectancy between the 95th and the 5th percentile in the US. In Argentina, analyses of intraurban variation in the risk of death[4] or in life expectancy among small areas showed wider variability in the metropolitan area of Buenos Aires (5.3 years for men) than in Córdoba.[26]

Differences in life expectancy between cities may be related to two key aspects. First, the use of different units of aggregation. Other articles[8] have use a broader definition of cities, encompassing peripheral areas around the core city that are often poorer, as compared to urban neighborhoods. However, if anything, using smaller units should lead to wider variability. Second, Córdoba may actually have narrower inequalities in mortality, i.e., gaps between 10th and 90th percentile. One would expect that cities like Córdoba with comparably less economic spatial segregation would have narrower differences in life expectancy at birth than those with more economic segregation (e.g. Buenos Aires). Indeed, gaps in life expectancy across neighborhoods in US cities have been strongly linked to levels of segregation[32]. Overall, there has been a trend from the 20th century of reductions in life expectancy gap across regions in Argentina,[33] but to date little is known about the intra-urban distribution in life expectancy, one of the most commonly used indicators to measure health.[34]

Additionally, we found that life expectancy at birth was geographically patterned, with higher life expectancy in the core and northwestern parts of the city, for both men and women, along with the southwestern part of the city for men. This suggested that variability in life expectancy was not random in Córdoba. Indeed, the city of Córdoba has an important socioeconomic residential segregation[35] that matched the spatial pattern of life expectancy: while the north-east-south periphery of the city predominately includes residential areas with poverty segregation, the central core and the northwest corridor are characterized by a greater concentration of wealth. There has been growing spatial concentration and social isolation of households with lower socioeconomic position as a consequence of public housing policies implemented in recent decades in the city of Córdoba. Particularly, relocations from informal settlements to large housing complexes built in the poor and semi-rural periphery of the city have been implemented by the provincial government between 2003 and 2007.[35,36] In addition, our findings are consistent with the significant intraurban inequalities in life expectancy recently described within Latin American cities.[6,7] The spatial pattern in Córdoba by which life expectancy was highest in the core and the northwest was more clearly

For peer review only - http://bmjopen.bmj.com/site/about/guidelines.xhtml

defined for women, while we also found higher life expectancy in the southern periphery among men. Previous research showed a similar core-periphery divide[8] and a north-to-south pattern[11] in Buenos Aires, which can be explained to some extent by the spatial patterning of socioeconomic deprivation. Our findings emphasize the need to address spatial variation in health within cities in further studies in the local context.

We also explored factors that might explain the heterogeneity in life expectancy across small areas. We found positive associations between improved socioeconomic characteristics of small areas and life expectancy at birth in both men and women, suggesting that the described heterogeneity in life expectancy might be driven by the socioeconomic features of small areas. All factors including educational attainment, school attendance, water access, overcrowding, unemployment, and the composite index were significantly associated with life expectancy at birth in the expected direction. These associations were stronger among men, similar to what was observed for the city of Buenos Aires,[26] although the magnitude of the associations between socioeconomic characteristics of small areas and life expectancy are lower in Córdoba compared to Buenos Aires.[26] Our findings are also consistent with other research in Argentina describing a negative association between an area-level deprivation index and life expectancy at birth in 32 urban agglomerations,[18] and a positive association between mortality rate and unmeet basic needs in Departamentos of the “Pampeana” region.[37] Furthermore, these results are consistent with factors associated with within-city inequalities in life expectancy in six large Latin American cities.[8]

Beyond the importance of individual[38] or household[25] socioeconomic status in predicting life expectancy, better social characteristics of small areas have been previously linked to lower mortality[4] and to higher life expectancy[26] in Argentina. Other characteristics of sub-city units such as higher levels of education, water access and sanitation and less overcrowding,[7] or higher area-level greenness[6] were also associated with higher life expectancy in Latin American cities. A similar relationship between increasing small area deprivation and decreasing life expectancy was previously described in New Zealand.[39] Residents of socially disadvantaged urban areas have to face the social and health consequences of a deprived environment, often surrounded by areas and individuals with similar socioeconomic needs [40], and deprived areas usually have less resources and services for health promotion, prevention and assistance.[3,7,41,42] This might exacerbate population morbidity and mortality burdens in urban areas with worse social conditions.[29]

This study adds to others[4,8,26] that characterized the geographic patterning and variation of mortality and life expectancy within large cities in Argentina by implementing small-area estimation approaches to investigate the distribution of unequal effects in small areas. Our work has several strengths. First, it represents the first estimation of life expectancy in small areas within the second largest city of Argentina, exhibiting the heterogeneity of life expectancy at birth within the city, and providing a description of what socioeconomic characteristics drive this heterogeneity. Second, this study employs a Bayesian approach that draws strength and smooths estimates from surrounding small areas and from the overall structure of the mortality schedule. Third, we estimated gender-specific life expectancy at birth, and linked mortality records to standardized socioeconomic data of small areas. While some Latin American countries have vital registration records with incomplete registration, this is less of a concern in Argentina.[7,43,44] Fourth, we examined these associations by means of univariate models that acknowledge uncertainty around the estimates of life expectancy by applying multiple imputation type techniques. Finally, in a sensitivity analysis, we considered potential biases in the estimation of infant mortality, by analyzing life expectancy at ages 20, 40, and

For peer review only - http://bmjopen.bmj.com/site/about/guidelines.xhtml

60. Their distribution showed similar patterns to those reported for life expectancy at birth, suggesting that our estimates of life expectancy at birth are reliable and robust to those factors.

Among the limitations of our study was the relatively high proportion of records that had low georeferencing accuracy, i.e., that were georeferenced to the street level. It was likely that these records were not randomly distributed. In fact, low georeferencing accuracy seemed to impact mostly small areas in the periphery. We decided to include these records in the study by assigning them to their most likely small area (e.g., using the center point of a street or area they were georeferenced to). This led to potentially more accurate but lower life expectancy estimates. We also needed to apply spatial and age smoothing to address the small-area estimation of mortality rates. This smoothing may have reduced inequalities, as reported in previous research.[8] We also had to combine population projections at the city level with 2010 census data, the only available data at the small area. If the population age or sex structure changed over time differentially by area, our estimates may be biased. Relatedly, social characteristics of small areas were retrieved from the same 2010 census, and were not aligned with the years for which death records were obtained (2015-2018). We therefore assumed that social characteristics were relatively stable across the years examined.

Conclusion

In summary, our study provided the first characterization of the heterogeneity in mortality and life expectancy in small areas in the city of Córdoba (Argentina), suggesting that the variability in life expectancy is not random and is potentially driven by social features of the small areas. We generate local evidence that is relevant for the targeting of health policies aimed to reduce health inequities.

Authors' contributions

SRL, NT, UB, KM and AVDR, conceived the study. BA and HQ did the statistical analyses. SRL, NT, and BA drafted the first version of the manuscript. SRL, NT, KM, ADQ, GEA and AVDR participated in or supported data collection. All authors participated in the interpretation of results and approved the final version of the manuscript.

Funding

The Salud Urbana en América Latina (SALURBAL)/ Urban Health in Latin America project is funded by the Wellcome Trust [205177/Z/16/Z]. NT was awarded by the European Union's Horizon 2020 research and innovation programme with the Marie Sklodowska-Curie grant agreement N◦ 89102. UB was supported by the Office of the Director of the National Institutes of Health under award number DP5OD26429. SRL gratefully acknowledge the financial support by the 'Agencia Nacional de Promoción Científica y Tecnológica' (ANPCyT) of Argentina, Project 'PICT CONICET 2018-01395'.

Competing interest statement

We declare no competing interests.

Role of funding source

For peer review only - http://bmjopen.bmj.com/site/about/guidelines.xhtml

The funder of the study had no role in study design, data collection, data analysis, data interpretation, or writing of this article.

Ethics approval statement

The SALURBAL study protocol was approved by the Drexel University Institutional Review Board with ID #1612005035 and by appropriate site-specific IRBs.

Data sharing statement

When the data can be made public without violating confidentiality -once the SALURBAL project ends-, it will be placed in a public repository as required by the funder (Wellcome Trust).

Acknowledgements

The authors acknowledge the contribution of all SALURBAL project team members. For more information on SALURBAL and to see a full list of investigators see https://drexel.edu/lac/salurbal/team/. We would like to thank Steve Melly for his help in creating the maps for this study. We thank Eugenia Peisino and Alicia Díaz for their help in accessing to the mortality data.

References

1 de Snyder VNS, Friel S, Fotso JC, *et al.* Social conditions and urban health inequities: Realities, challenges and opportunities to transform the urban landscape through research and action. *Journal of Urban Health* 2011;**88**:1183–93. doi:10.1007/s11524-011-9609-y

2 Borrell C, Pons-Vigués M, Morrison J, *et al.* Factors and processes influencing health inequalities in urban areas. *Journal of Epidemiology and Community Health* 2013;**67**:389–91. doi:10.1136/jech-2012-202014

3 Diez Roux A v, Mair C. Neighborhoods and health. *Annals of the New York Academy of Sciences* 2010;**1186**:125–45. doi:10.1111/j.1749-6632.2009.05333.x

4 Diez Roux AV, Green Franklin T, Alazraqui M, *et al.* Intraurban Variations in Adult Mortality in a Large Latin American City. *Journal of Urban Health: Bulletin of the New York Academy of Medicine* 2007;**84**.

5 Geraldi J. *UN HABITAT, State of the World's Cities 2010/2011: Cities for All, Bridging the Urban Divide*. London: : UN-HABITAT 2010. doi:10.4000/rccs.4470

6 Moran MR, Bilal U, Dronova I, *et al.* Greenness and education inequalities in life expectancy in Latin American cities: an ecological study. *The Lancet Planetary Health* 2021;**5**:S19. doi:10.1016/S2542-5196(21)00103-0

7 Bilal U, Hessel P, Perez-Ferrer C, Michael Y, Alfaro T, Tenorio-Mucha J, de Friche AA, Pina MF, Vives A, Quick H, Alazraqui M, Rodriguez D, Miranda JJ, Diez-Roux AV and the S study team. Life

For peer review only - http://bmjopen.bmj.com/site/about/guidelines.xhtml

expectancy and mortality in 363 cities of Latin America. *Nature Medicine* 2021;**27**:463–70. doi:https://doi.org/10.1038/s41591-020-01214-4

8 Bilal U, Alazraqui M, Caiaffa WT, *et al.* Inequalities in life expectancy in six large Latin American cities from the SALURBAL study: an ecological analysis. *The Lancet Planetary Health* 2019;**3**:e503–10. doi:https://doi.org/10.1016/S2542-5196(19)30235-9

9 Pou SA, Tumas N, Sánchez Soria D, *et al.* Large-scale societal factors and noncommunicable diseases: Urbanization, poverty and mortality spatial patterns in Argentina. *Applied Geography* 2017;**86**:32–40. doi:http://dx.doi.org/10.1016/j.apgeog.2017.06.022

10 de Maio FG. Ecological analysis of the health effects of income inequality in Argentina. *Public Health* 2008;**122**:487–96. doi:http://dx.doi.org/10.1016/j.puhe.2007.09.006

11 Macchia A, Mariani J, Nul D, *et al.* Social inequity, place of residence and all-cause premature death in Argentina. *Revista Argentina de Cardiología* 2016;**84**:108–13. doi:10.7775/RAC.V84.I2.8267

12 Snyder RE, Jaimes G, Riley LW, *et al.* A Comparison of social and spatial determinants of health between formal and informal settlements in a large metropolitan setting in Brazil. *Journal of Urban Health 2013 91:3* 2013;**91**:432–45. doi:10.1007/S11524-013-9848-1

13 James C. Featured Graphic. Lives on the Line: Mapping Life Expectancy along the London Tube Network: *Environment and Planning A: Economy and Space* 2012;**44**:1525–8. doi:10.1068/A45341

14 Quistberg DA, Diez Roux A v., Bilal U, *et al.* Building a Data Platform for Cross-Country Urban Health Studies: the SALURBAL Study. *Journal of Urban Health* 2019;**96**:311–37. doi:10.1007/s11524-018-00326-0

15 INDEC. Censo Nacional de Población, Hogares y Viviendas 2010 [National Census of Population, Households and housing 2010]. 2010.https://www.indec.gob.ar/indec/web/Nivel4-Tema-2-41-135

16 Rizzi S, Gampe J, Eilers PHC. Efficient Estimation of Smooth Distributions from Coarsely Grouped Data. *American Journal of Epidemiology* 2015;**182**:138–47. doi:10.1093/aje/kwv020

17 Durán RJ, Condorí MÁ. Deprivation Index for Small Areas Based on Census Data in Argentina. *Social Indicators Research* 2019;**141**:331–63. doi:10.1007/s11205-017-1827-6

18 Ivaldi E, Parra Saiani P, Primosich JJ, *et al.* Health and Deprivation: A New Approach Applied to 32 Argentinian Urban Areas. *Social Indicators Research* 2020;**151**:155–79. doi:10.1007/s11205-020-02369-w

19 Gonzaga MR, Schmertmann CP. Estimating age- and sex-specific mortality rates for small areas with topals regression: An application to Brazil in 2010. *Revista Brasileira de Estudos de Populacao* 2016;**33**:629–52. doi:10.20947/S0102-30982016c0009

For peer review only - http://bmjopen.bmj.com/site/about/guidelines.xhtml

20 Schmertmann CP, Gonzaga MR. Bayesian Estimation of Age-Specific Mortality and Life Expectancy for Small Areas With Defective Vital Records. *Demography 2018 55:4* 2018;**55**:1363–88. doi:10.1007/S13524-018-0695-2

21 Lunn DJ, Thomas A, Best N, *et al.* WinBUGS — a Bayesian modelling framework: concepts, structure, and extensibility. *Statistics and Computing* 2000;**10**:325–337.

22 Sturtz S, Ligges U, Gelman A. R2WinBUGS: A Package for Running WinBUGS from R. *Journal of Statistical Software* 2005;**12**:1–16.

23 Riffe T, Aburto JM, Alexander M, Fennell S, Kashnitsky I, Pascariu M GP. DemoTools: An R package of tools for aggregate demographic analysis. 2019.https://github.com/timriffe/DemoTools/

24 Modig K, Rau R, Ahlbom A. Life expectancy: what does it measure? *BMJ Open* 2020;**10**:e035932. doi:10.1136/bmjopen-2019-035932

25 Merlo J, Viciana-Fernandez FJ, Ramiro-Farinas D, *et al.* Bringing the individual back to small-area variation studies: A multilevel analysis of all-cause mortality in Andalusia, Spain. *Social Science & Medicine* 2012;**75**:1477–87.

26 Trotta A, Diez Roux A, Bilal U, *et al.* Inequity and life expectancy in Buenos Aires, 2015-2017: a small area estimation approach. *[Manuscript submitted for publication]* 2022.

27 Tumas N, Rodríguez S, Bilal U, *et al.* Urban social determinants of non-communicable diseases risk factors in Argentina. *Health & Place* 2021;:102611.https://doi.org/10.1016/j.healthplace.2021.102611

28 Boing AF, Boing AC, Cordes J, *et al.* Quantifying and explaining variation in life expectancy at census tract, county, and state levels in the United States. *Proceedings of the National Academy of Sciences* 2020;**117**:17688–94. doi:10.1073/PNAS.2003719117

29 Szwarcwald CL, Mota JC da, Damacena GN, *et al.* Health Inequalities in Rio de Janeiro, Brazil: Lower Healthy Life Expectancy in Socioeconomically Disadvantaged Areas. *American Journal of Public Health* 2011;**101**:517–23. doi:10.2105/AJPH.2010.195453

30 Badaloni C, Michelozzi P, Davoli M. Spatial and temporal variation of life expectancy at birth by socioeconomic position and district in Rome for the years 2006-2017. *Epidemiologia e prevenzione* 2020;**44**:342–8. doi:10.19191/EP20.5-6.P342.009

31 National Records of Scotland. Life Expectancy in Scottish Areas 2016-2018. 2019.

32 Spoer BR, Juul F, Hsieh PY, *et al.* Neighborhood-level Asian American Populations, Social Determinants of Health, and Health Outcomes in 500 US Cities. *Ethnicity & Disease* 2021;**31**:433–44. doi:10.18865/ED.31.3.433

33 Grushka C. Casi un siglo y medio de mortalidad en la Argentina…. *Revista Latinoamericana de Población* 2014;**8**:93–118. doi:10.31406/RELAP2014.V8.I2.N15.4

34 Eckert S, Kohler S. Urbanization and health in developing countries: a systematic review. *World Health & Population* 2014;**15**:7–20. doi:10.12927/WHP.2014.23722

For peer review only - http://bmjopen.bmj.com/site/about/guidelines.xhtml

35 Molinatti F. Segregación residencial socioeconómica en la ciudad de Córdoba (Argentina): Tendencias y patrones espaciales. *Revista INVI* 2013;**28**:61–94.

36 Marengo C. Estrategias habitacionales en los bordes urbanos. *Proyección* 2002;**1**.

37 Sacco N, Williams I, L. Queiroz B. Estimaciones de esperanza de vida al nacer en áreas menores de la región pampeana. *Revista Brasileira de Estudos de População* 2021;**38**:1–41. doi:10.20947/s0102-3098a0160

38 Stringhini S, Carmeli C, Jokela M, *et al.* Socioeconomic status and the 25 x 25 risk factors as determinants of premature mortality: a multicohort study and meta-analysis of 1.7 million men and women. *The Lancet* 2017;**389**:1229–37. doi:http://dx.doi.org/10.1016/S0140-6736(16)32380-7

39 Tobias MI, Cheung J. Monitoring health inequalities: life expectancy and small area deprivation in New Zealand. *Population Health Metrics* 2003;**1**:1–11. doi:10.1186/1478-7954-1-2

40 Stafford M, Marmot M. Neighbourhood deprivation and health: does it affect us all equally? *International Journal of Epidemiology* 2003;**32**:357–66. doi:10.1093/IJE/DYG084

41 Boylan JM, Robert SA. Neighborhood SES is particularly important to the cardiovascular health of low SES individuals. *Social Science & Medicine* 2017;**188**:60–8. doi:10.1016/j.socscimed.2017.07.005

42 Arcaya MC, Tucker-Seeley RD, Kim R, *et al.* Research on neighborhood effects on health in the United States: a systematic review of study characteristics. *Social Science & Medicine* 2016;**168**:16–29.

43 Peralta A, Benach J, Borrell C, *et al.* Evaluation of the mortality registry in Ecuador (2001–2013) – social and geographical inequalities in completeness and quality. *Population Health Metrics* 2019;**17**:1–12. doi:10.1186/S12963-019-0183-Y

44 Adair T, Lopez AD. Estimating the completeness of death registration: An empirical method. *PLOS ONE* 2018;**13**:e0197047. doi:10.1371/JOURNAL.PONE.0197047

For peer review only

For peer review only - http://bmjopen.bmj.com/site/about/guidelines.xhtml

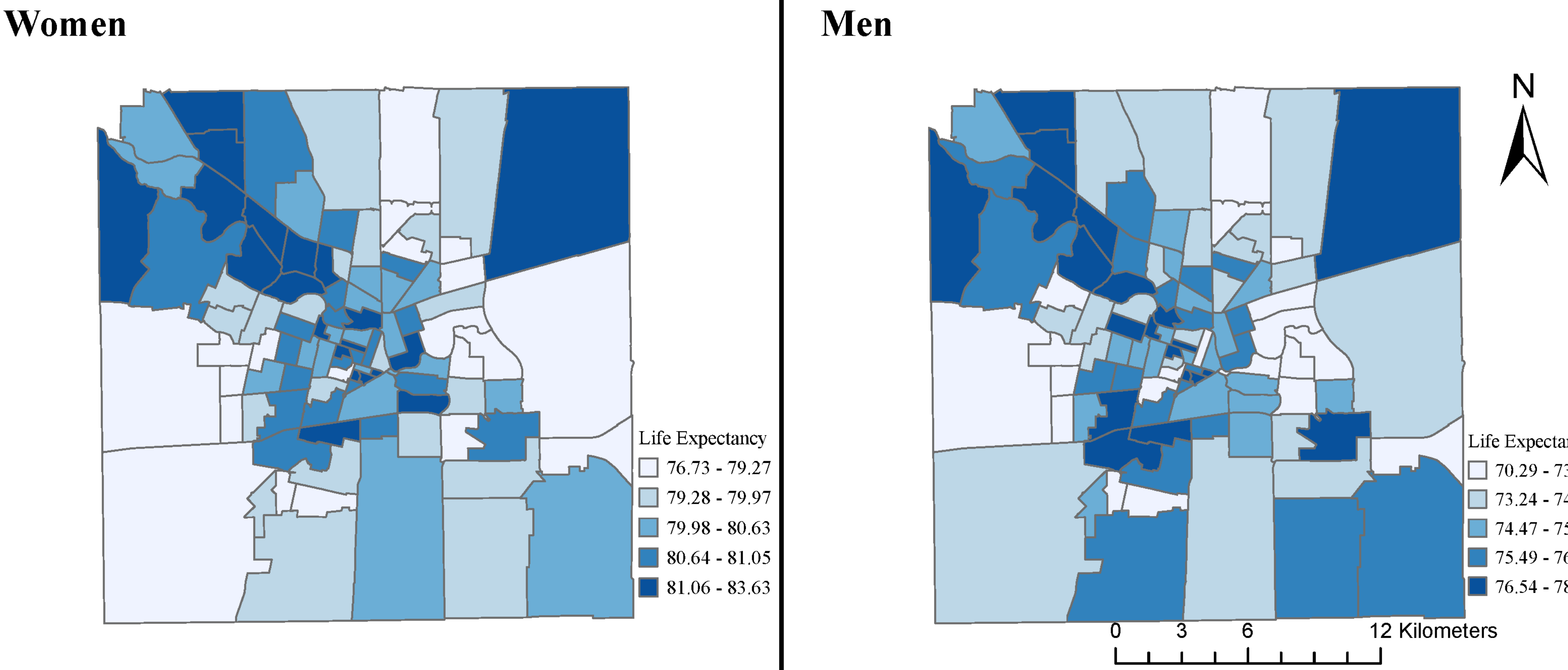

For peer review only - http://bmjopen.bmj.com/site/about/guidelines.xhtml

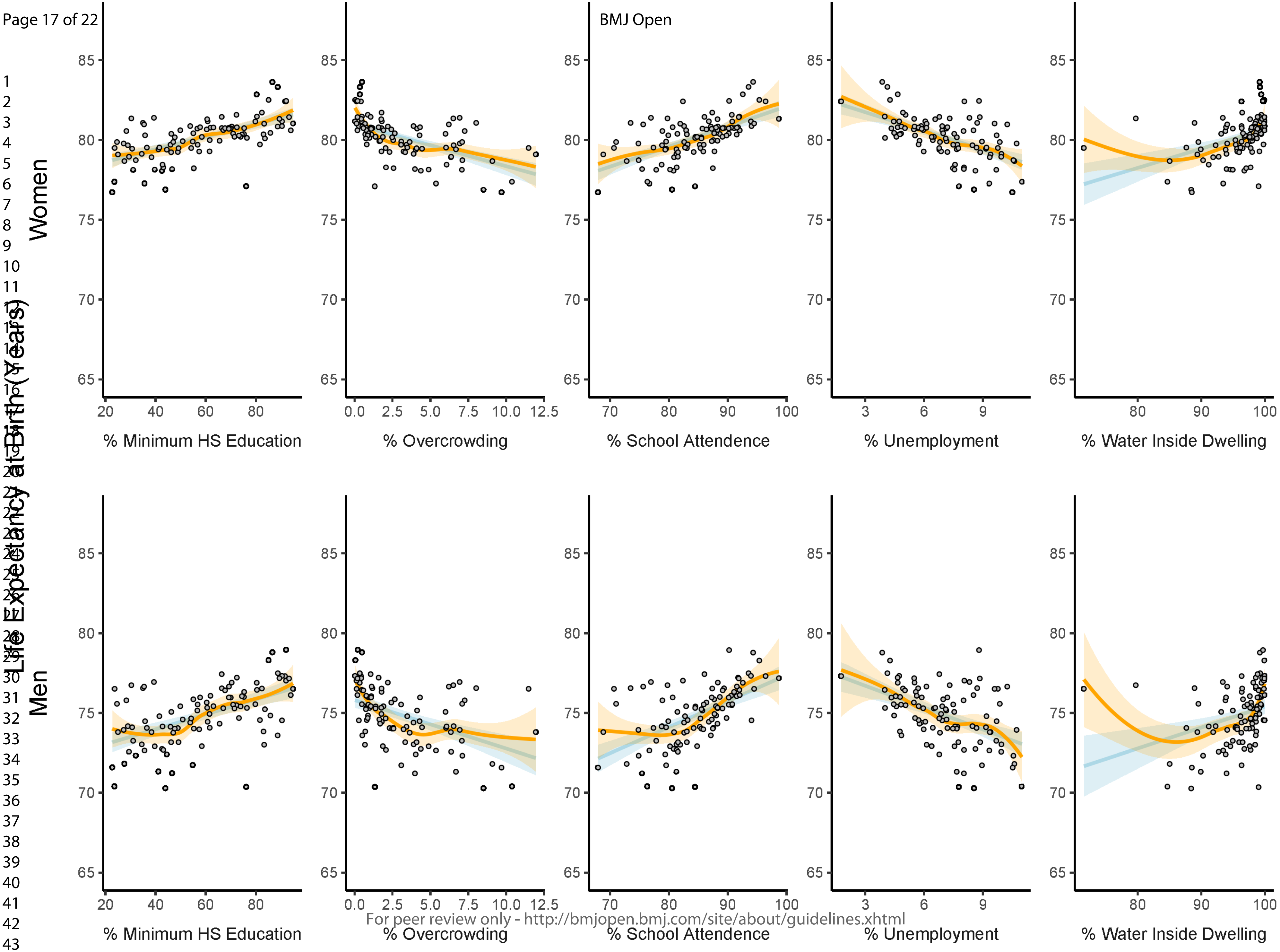
Life Expectancy at Birth (Years)
Women
Men
85
80
75
70
65
% Minimum HS Education
20
40
60
80
% Overcrowding
0.0
2.5
5.0
7.5
10.0
12.5
% School Attendence
70
80
90
100
% Unemployment
3
6
9
% Water Inside Dwelling
80
90
100

Supplementary material

Modelling the mortality rate

We employ the Bayesian adaptation of the tool for projecting age patterns using linear splines method (TOPALS) which incorporates spatial smoothing in small areas.[19]

For age $a\,\{a = 0,1,2,\ldots,85\}$ and small area $i\,\{i = 1,2,\ldots,99\}$ with corresponding population n_{ia}, we assume that the number of deaths comes from a Poisson distribution with underlying rate λ_{ia}.

$$y_{ia} | \lambda_{ia} \sim Pois\,(n_{ia}\,\lambda_{ia})$$

We further assume that the vector of log mortality rates small areas i is,

$$log(\boldsymbol{\lambda}_{i.}) = log(\boldsymbol{\lambda}^{*}) + \boldsymbol{B}\,\boldsymbol{\beta}_{\boldsymbol{k}.},$$

Where $\boldsymbol{\lambda}_{\boldsymbol{i}.}$ is the vecor with elements λ_{ia} representing age-specific mortality rates in small areas, $\boldsymbol{\lambda}^{*}$ is the vector of standard mortality schedule (i.e., the smoothed city-level rates), $\boldsymbol{B}$ is a matrix of constants of size 86×7 in which each column is a linear B-spline basis function, and $\boldsymbol{\beta}_{\boldsymbol{k}.}$ is a vector of parameters with elements β_{ik} representing offsets to the standard schedule.
We define knots at ages $t_0,\ldots,t_6$ = (0, 1, 10, 20, 40, 70, 85). For ages a in {0, 1, 2, ..., 85} and columns k in {1,...,7} the basis functions in $\boldsymbol{B}$ are:

$$\frac{a - t_{k-1}}{t_k - t_{k-1}}\ if\ t_{k-1} \le a \le t_k;$$

$$\frac{t_{k+1} - a}{t_{k+1} - t_k}\ if\ t_k \le a \le t_{k+1};$$

$$0\ \ otherwise.$$

We further decompose the β_{ik} into the intercepts at each knot β_{0k}; the spatial random effects, z_{ik} ; and unstructured random effects (ϕ_{ik}) that vary by knot age and area.

$$\beta_{ik} = \beta_{0k} + z_{ik} + \phi_{ik}\,.$$

We assign the unstructured, non-spatial random effect (ϕ_{ik}) an exchangeable zero-mean normal prior with the knot-specific variance $\sigma^2_{ns;\,k}$. The variance parameter in turn receives the uninformative inverse gamma hyper-prior with the shape and rate parameter of 1 and 0.01, respectively.
That is,

$$\phi_{ik} \sim Normal\,(0, \sigma^2_{ns;\,k})$$
$$\sigma^2_{ns;\,k} \sim Inverse\ Gamma\,(1, 0\cdot 01)$$

For the intercept β_{0k}, we assign a vague normal prior with mean 0 and variance of 1,000. That is,

$$\beta_{0k} \sim Normal\,(0, 1000)$$

For the spatial random effect β_{ik}, we assign the intrinsic conditional autoregressive (ICAR) prior distribution[45] for each knots, k. We define areas i and j as neighbors if they share one or more common vertex between boundaries, commonly referred to as Queen's contiguity.
For any given knot and for each area i, the conditional expected value of z_{ik} is the mean of its neighboring areas, and the variance of z_{ik} is inversely proportional to the number of neighbors for that area, m_i.
If we drop the subscript for knot here, we can denote the CAR distribution as:

$$z_i | z_{-i}, \boldsymbol{W},\ \sigma^2_z \sim Normal\,(\,\bar{z}_i, \frac{\sigma^2_z}{m_i}),$$

where

$$\bar{z}_i = \sum_{j,j\neq i} \frac{w_{i,j}\, z_j}{m_i}$$

and $W = [w_{i,j}]$ is 99 × 99 adjacency matrix with elements $w_{i,j} = 1$ if areas i and j are neighbors and 0 otherwise. We complete the prior specification by assigning a weakly informative inverse gamma prior (1, 0·14) for the variance of CAR random effects.

For peer review only - http://bmjopen.bmj.com/site/about/guidelines.xhtml

Figure S1. Log mortality rates due to all causes for women and men. Córdoba, 2015-2018.

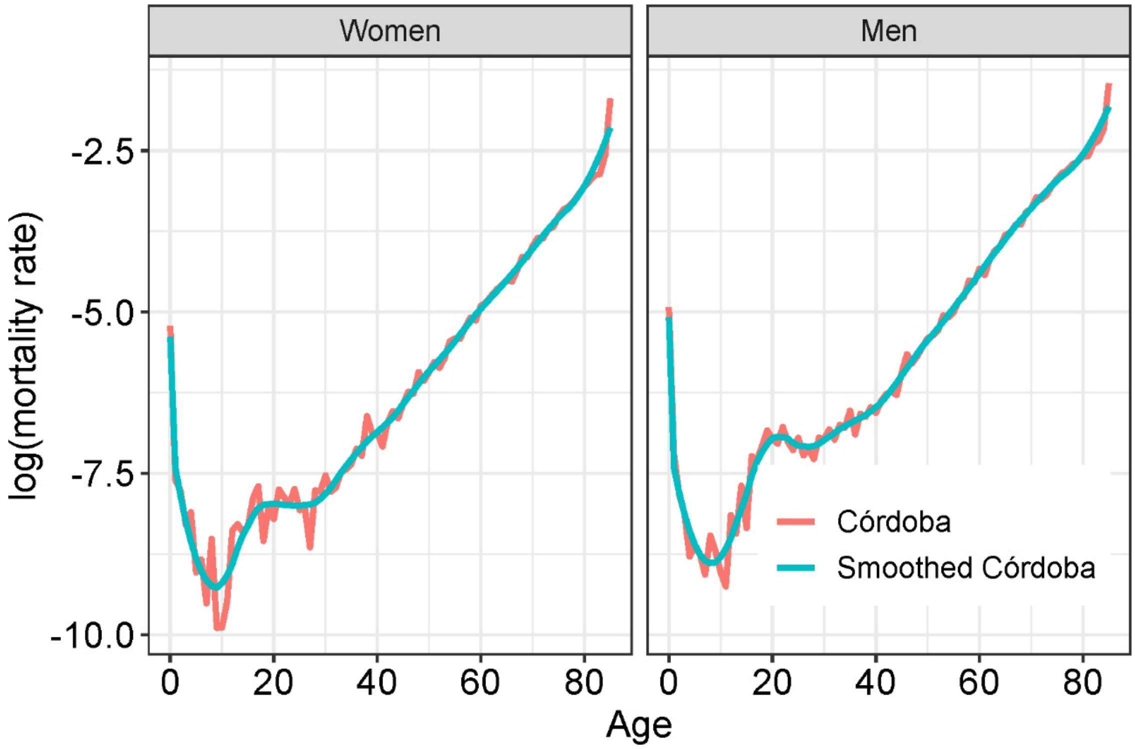

Footnote: The Loess-smoothed line served as the standard schedule in the analysis.

For peer review only

For peer review only - http://bmjopen.bmj.com/site/about/guidelines.xhtml

Figure S2. Life expectancy at birth (95% CI) among women and men, by small area. Córdoba, 2015-2018.

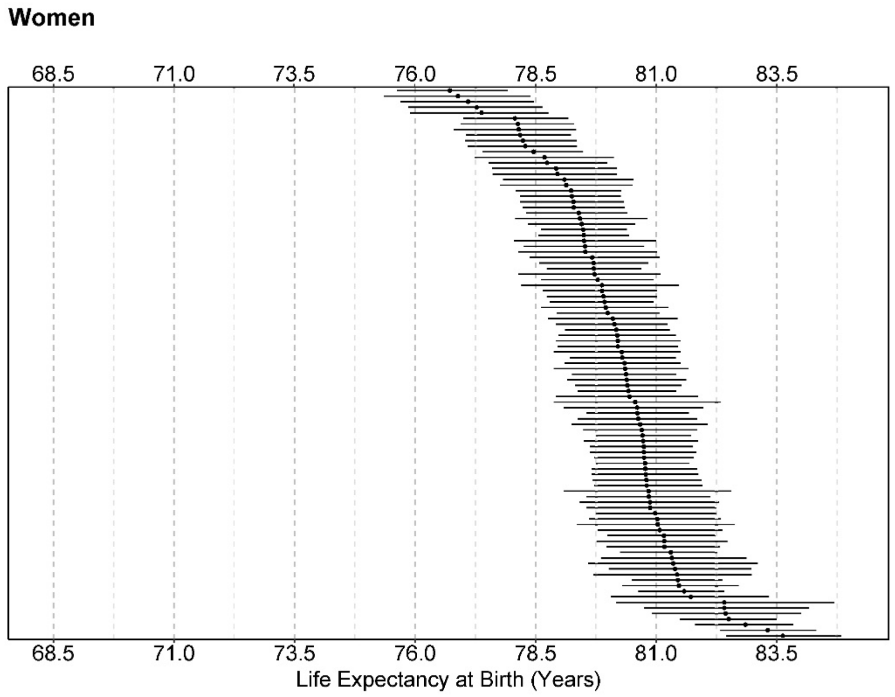

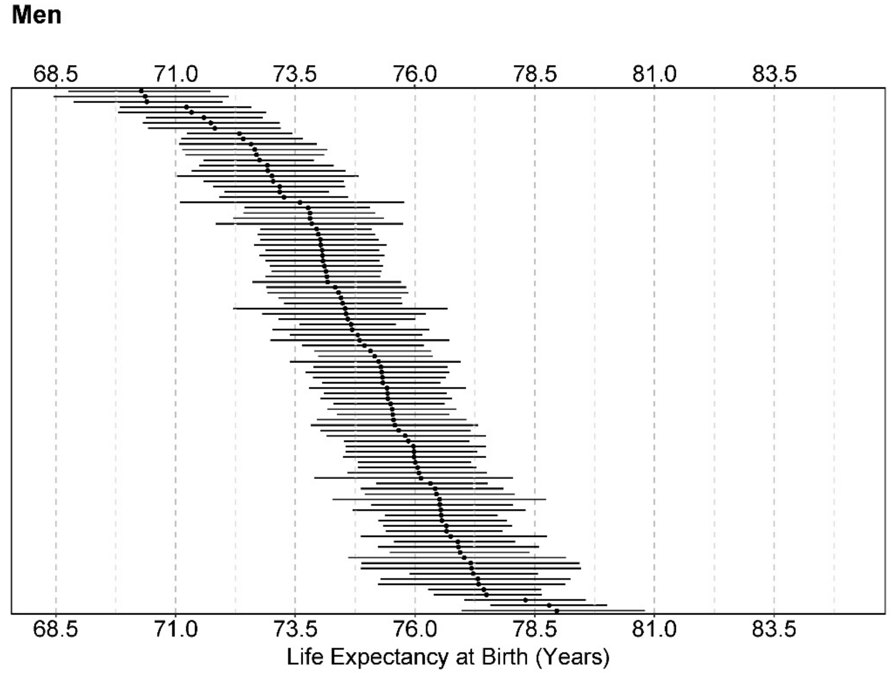

For peer review only - http://bmjopen.bmj.com/site/about/guidelines.xhtml

Figure S3. Spatial distribution of life expectancy at birth, 20, 40 and 60 years in women and men in the 99 small areas of city of Córdoba, 2015-2018.

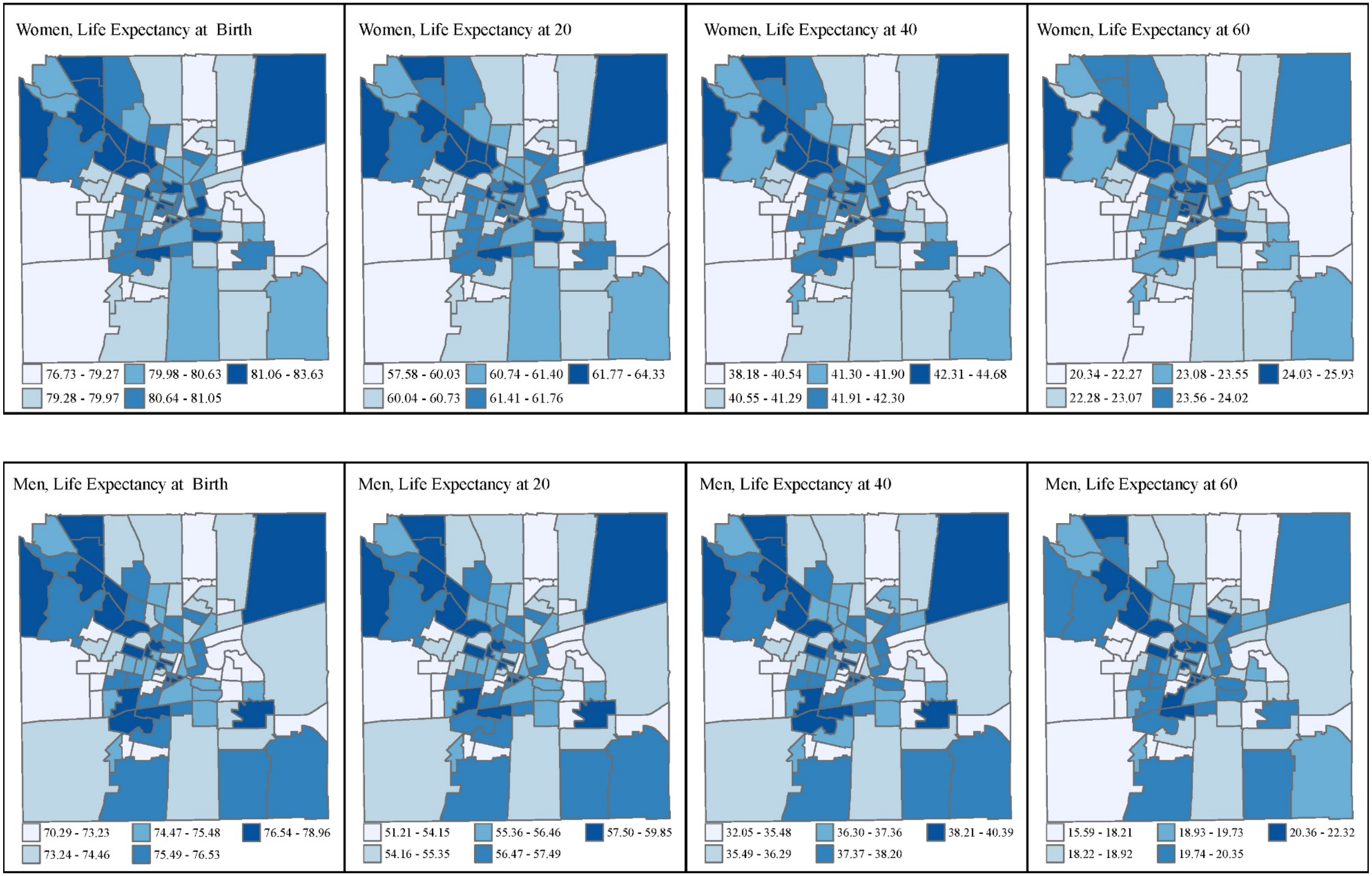

STROBE Statement—Checklist of items that should be included in reports of ***cross-sectional studies***

	Item No	Recommendation	Page No
**Title and abstract**	1	(*a*) Indicate the study's design with a commonly used term in the title or the abstract	1
		(*b*) Provide in the abstract an informative and balanced summary of what was done and what was found	1
**Introduction**			
Background/rationale	2	Explain the scientific background and rationale for the investigation being reported	2
Objectives	3	State specific objectives, including any prespecified hypotheses	2
**Methods**			
Study design	4	Present key elements of study design early in the paper	2
Setting	5	Describe the setting, locations, and relevant dates, including periods of recruitment, exposure, follow-up, and data collection	2
Participants	6	(*a*) Give the eligibility criteria, and the sources and methods of selection of participants	2
Variables	7	Clearly define all outcomes, exposures, predictors, potential confounders, and effect modifiers. Give diagnostic criteria, if applicable	3
Data sources/ measurement	8*	For each variable of interest, give sources of data and details of methods of assessment (measurement). Describe comparability of assessment methods if there is more than one group	3
Bias	9	Describe any efforts to address potential sources of bias	3
Study size	10	Explain how the study size was arrived at	3
Quantitative variables	11	Explain how quantitative variables were handled in the analyses. If applicable, describe which groupings were chosen and why	3
Statistical methods	12	(*a*) Describe all statistical methods, including those used to control for confounding	3-4
		(*b*) Describe any methods used to examine subgroups and interactions	3-4
		(*c*) Explain how missing data were addressed	3
		(*d*) If applicable, describe analytical methods taking account of sampling strategy	
		(*e*) Describe any sensitivity analyses	3
**Results**			
Participants	13*	(a) Report numbers of individuals at each stage of study—eg numbers potentially eligible, examined for eligibility, confirmed eligible, included in the study, completing follow-up, and analysed	4-5
		(b) Give reasons for non-participation at each stage	
		(c) Consider use of a flow diagram	
Descriptive data	14*	(a) Give characteristics of study participants (eg demographic, clinical, social) and information on exposures and potential confounders	4-5
		(b) Indicate number of participants with missing data for each variable of interest	
Outcome data	15*	Report numbers of outcome events or summary measures	4-5
Main results	16	(*a*) Give unadjusted estimates and, if applicable, confounder-adjusted estimates and their precision (eg, 95% confidence interval). Make clear which confounders were adjusted for and why they were included	5-6

For peer review only

		(*b*) Report category boundaries when continuous variables were categorized	
		(*c*) If relevant, consider translating estimates of relative risk into absolute risk for a meaningful time period	
Other analyses	17	Report other analyses done—eg analyses of subgroups and interactions, and sensitivity analyses	5
**Discussion**			
Key results	18	Summarise key results with reference to study objectives	6
Limitations	19	Discuss limitations of the study, taking into account sources of potential bias or imprecision. Discuss both direction and magnitude of any potential bias	8
Interpretation	20	Give a cautious overall interpretation of results considering objectives, limitations, multiplicity of analyses, results from similar studies, and other relevant evidence	9
Generalisability	21	Discuss the generalisability (external validity) of the study results	9
**Other information**			
Funding	22	Give the source of funding and the role of the funders for the present study and, if applicable, for the original study on which the present article is based	9

*Give information separately for exposed and unexposed groups.

Note: An Explanation and Elaboration article discusses each checklist item and gives methodological background and published examples of transparent reporting. The STROBE checklist is best used in conjunction with this article (freely available on the Web sites of PLoS Medicine at http://www.plosmedicine.org/, Annals of Internal Medicine at http://www.annals.org/, and Epidemiology at http://www.epidem.com/). Information on the STROBE Initiative is available at www.strobe-statement.org.

For peer review only

BMJ Open

Intraurban socioeconomic inequalities in life expectancy: a population-based cross-sectional analysis in the city of Córdoba, Argentina (2015-2018)

Journal:	*BMJ Open*
Manuscript ID	bmjopen-2022-061277.R1
Article Type:	Original research
Date Submitted by the Author:	15-Aug-2022

Complete List of Authors:	Rodríguez López, Santiago ; Consejo Nacional de Investigaciones Cientificas y Tecnicas, Centro de Investigaciones y Estudios sobre Cultura y Sociedad; Universidad Nacional de Córdoba Facultad de Ciencias Exactas Físicas y Naturales, Cátedra de Antropología<br>Tumas, Natalia; Universitat Pompeu Fabra, Research Group on Health Inequalities, Environment, and Employment Conditions Network (GREDS-EMCONET), Department of Social and Political Science; Consejo Nacional de Investigaciones Cientificas y Tecnicas, Centro de Investigaciones y Estudios sobre Cultura y Sociedad<br>Bilal, Usama; Drexel University Dornsife School of Public Health, Urban Health Collaborative; Drexel University Dornsife School of Public Health, Department of Epidemiology and Biostatistics<br>Moore, Kari; Drexel University School of Public Health, Urban Health Collaborative<br>Acharya, Binod; Drexel University Dornsife School of Public Health, Urban Health Collaborative<br>Quick, Harrison; Drexel University Dornsife School of Public Health, Urban Health Collaborative; Drexel University Dornsife School of Public Health, Department of Epidemiology and Biostatistics<br>Quistberg, Alex; Drexel University Dornsife School of Public Health, Urban Health Collaborative; Drexel University Dornsife School of Public Health, Department of Environmental and Occupational Health<br>Acevedo, Gabriel; Universidad Nacional de Córdoba Facultad de Ciencias Médicas, Cátedra de Medicina Preventiva y Social<br>Diez Roux, Ana V.; Drexel University Dornsife School of Public Health, Urban Health Collaborative; Drexel University Dornsife School of Public Health, Department of Epidemiology and Biostatistics

**Primary Subject Heading**:	Public health
Secondary Subject Heading:	Research methods, Epidemiology
Keywords:	PUBLIC HEALTH, SOCIAL MEDICINE, EPIDEMIOLOGY

I, the Submitting Author has the right to grant and does grant on behalf of all authors of the Work (as defined in the below author licence), an exclusive licence and/or a non-exclusive licence for contributions from authors who are: i) UK Crown employees; ii) where BMJ has agreed a CC-BY licence shall apply, and/or iii) in accordance with the terms applicable for US Federal Government officers or employees acting as part of their official duties; on a worldwide, perpetual, irrevocable, royalty-free basis to BMJ Publishing Group Ltd ("BMJ") its licensees and where the relevant Journal is co-owned by BMJ to the co-owners of the Journal, to publish the Work in this journal and any other BMJ products and to exploit all rights, as set out in our licence.

The Submitting Author accepts and understands that any supply made under these terms is made by BMJ to the Submitting Author unless you are acting as an employee on behalf of your employer or a postgraduate student of an affiliated institution which is paying any applicable article publishing charge ("APC") for Open Access articles. Where the Submitting Author wishes to make the Work available on an Open Access basis (and intends to pay the relevant APC), the terms of reuse of such Open Access shall be governed by a Creative Commons licence – details of these licences and which Creative Commons licence will apply to this Work are set out in our licence referred to above.

Other than as permitted in any relevant BMJ Author's Self Archiving Policies, I confirm this Work has not been accepted for publication elsewhere, is not being considered for publication elsewhere and does not duplicate material already published. I confirm all authors consent to publication of this Work and authorise the granting of this licence.

For peer review only

Intraurban socioeconomic inequalities in life expectancy: a population-based cross-sectional analysis in the city of Córdoba, Argentina (2015-2018)

Santiago Rodríguez López*[1,2]; Natalia Tumas[1,3]; Usama Bilal[4,5]; Kari Moore[4]; Binod Acharya[4]; Harrison Quick[4,5]; D. Alex Quistberg[4,6]; Gabriel E. Acevedo[7]; Ana V. Diez Roux[4,5]

[1] Centro de Investigaciones y Estudios sobre Cultura y Sociedad, Consejo Nacional de Investigaciones Científicas y Técnicas y Universidad Nacional de Córdoba (CIECS, CONICET y UNC), Argentina.

[2] Cátedra de Antropología, Facultad de Ciencias Exactas, Físicas y Naturales, Universidad Nacional de Córdoba, Argentina.

[3] Research Group on Health Inequalities, Environment, and Employment Conditions Network (GREDS-EMCONET), Department of Social and Political Science, Universitat Pompeu Fabra, Barcelona, Spain.

[4] Urban Health Collaborative, Dornsife School of Public Health, Drexel University, Philadelphia, USA.

[5] Department of Epidemiology and Biostatistics, Dornsife School of Public Health, Drexel University, Philadelphia, USA.

[6] Department of Environmental and Occupational Health, Dornsife School of Public Health, Drexel University, Philadelphia, USA.

[7] Cátedra de Medicina Preventiva y Social, Facultad de Ciencias Médicas, Universidad Nacional de Córdoba, Argentina.

***Corresponding author:** Santiago Rodríguez López. Centro de Investigaciones y Estudios sobre Cultura y Sociedad (CIECS, CONICET y UNC), Av. Valparaíso s/n, Ciudad Universitaria, Córdoba, Argentina. C.P: 5000. +54 351 535 3769. e-mail: santiago.rodriguez@conicet.gov.ar/ santiago.rodriguez@unc.edu.ar

Keywords: life expectancy; neighborhoods; small-area estimation; Argentina; TOPALS

Word count: 3,787

For peer review only - http://bmjopen.bmj.com/site/about/guidelines.xhtml

Abstract

Objectives: To evaluate variability in life expectancy at birth in small areas, describe the spatial pattern of life expectancy, and examine associations between small-area socioeconomic characteristics and life expectancy in a mid-sized city of a middle-income country.
Design: Cross-sectional, using data from death registries (2015-2018) and socioeconomic characteristics data from the 2010 national population census.
Participants/setting: 40,898 death records in 99 small areas of the city of Córdoba, Argentina. We summarized variability in life expectancy at birth by using the difference between the 90th and 10th percentile of the distribution of life expectancy across small areas (P90-P10 gap) and evaluated associations with small-area socioeconomic characteristics by calculating a Slope Index of Inequality (SII) in linear regression.
Primary outcome: Life expectancy at birth.
Results: The median life expectancy at birth was 80.3 years in women (P90-P10 gap=3.2 years) and 75.1 years in men (P90-P10 gap=4.6 years). We found higher life expectancies in the core and northwest parts of the city, especially among women. We found positive associations between life expectancy and better small-area socioeconomic characteristics, especially among men. Mean differences in life expectancy between the highest versus the lowest decile of area characteristics in men (women) were 3.03 (2.58), 3.52 (2.56), and 2.97 (2.31) years for % adults with high school education or above, % persons aged 15-17 attending school, and % households with water inside the dwelling, respectively. Lower values of % overcrowded households and unemployment rate were associated with longer life expectancy: mean differences comparing the lowest versus the highest decile were 3.03 and 2.73 in men and 2.34 and 2.69 years in women, respectively.
Conclusion: Life expectancy is substantially heterogeneous and patterned by socioeconomic characteristics in a mid-sized city of a middle-income country, suggesting that small-area inequities in life expectancy are not limited to large cities or high-income countries.

Strengths and limitations

- The study had unique access to mortality data georeferenced to small areas.
- The Bayesian approach draws strength from surrounding small areas and from the overall structure of the mortality schedule allowing valid and reliable estimation even when data is sparse.
- The use of the Slope Index of Inequality (SII) takes advantage of the full distribution of socioeconomic variables but avoids excessive influence of extreme values.
- Excessive smoothing may have resulted in underestimated inequities.
- Mortality and census population and socioeconomic data were not exactly aligned in time, thus it was necessary to assume that the social characteristics of small areas, the population age, and sex structure were relatively stable across the years examined.

For peer review only - http://bmjopen.bmj.com/site/about/guidelines.xhtml

Introduction

The social context of daily life is linked to health outcomes and generates social inequalities in health.[1] Health inequalities tend to be large in urban areas because cities include areas of concentrated deprivation and poverty,[2,3] especially in large cities where spatial and social inequalities are combined.[4] Latin America is one of the most urbanized regions of the world with urbanization levels estimated to be in the order of 80 %.[5] It is also one of the most unequal regions in the world and is home to more than half of the thirty most unequal cities in the world.[6]

Although a number of studies have documented large inequities in mortality and life expectancy across neighborhoods within cities in high-income countries,[7–11] there has been very limited investigation of within-city heterogeneity in life expectancy in cities of lower- and middle-income countries. Indeed, some work has suggested that the educational patterning of adult mortality in middle-income countries can be different from that observed in high-income countries.[12]

Argentina, one of the most urbanized countries in Latin America, is a middle-income country with large social and health disparities.[13] However, evidence of intraurban health variations across small areas is still scarce. Prior research showed that higher income inequality at the provincial level was associated with lower life expectancy for both men and women.[14] Another study described significant associations between social inequity and premature death rates in departments of Argentina.[15] Diez Roux et al.[4] described substantial intraurban variation in the risk of death in the core area of the large urban agglomeration of Buenos Aires, finding similar patterns to another study examining life expectancy across the entire metropolitan area.[16] These studies are primarily focused on the city of Buenos Aires, but no studies of which we are aware have so far addressed variability and the social patterning of life expectancy at birth in smaller cities which may be more representative of a larger number of cities across the Latin American region.

The city of Córdoba, Argentina, provincial capital of 1.3 million residents, is emblematic of emerging cities across the region as a medium-sized city with fast urban growth. As is the case for other cities, generating local evidence to fill the knowledge gap on existing social inequities in life expectancy at birth in the city of Córdoba is relevant to informing policies to improve health conditions.[17] Examining these issues in Córdoba can both inform local policy while also provide a case study of an emerging medium-sized city in the region. Prior research has suggested substantial heterogeneity of life expectancy and mortality in Latin American cities[16,18], thus exploring these issues in small areas across heterogeneous cities is important for understanding the need to address them in different urban contexts and development levels, especially as much of the future urban growth in the region will likely be in cities like Córdoba.[19]

To assess the extent to which spatial patterning of life expectancy and its association with small-area socioeconomic characteristics extends beyond the largest urban agglomerations to other cities in middle-income countries, we examined small-area variations in life expectancy in the city of Córdoba. Our goals were to 1) evaluate the variability in life expectancy at birth in small areas of the city of Córdoba for the 2015-2018 period; 2) describe the spatial patterning of life expectancy at birth and; 3) examine the association between small area socioeconomic characteristics and life expectancy at birth. We hypothesized that (1) there is considerable variability of life expectancy at birth across small areas of Córdoba; (2) there is lower life expectancy in the periphery of the city of Córdoba as

For peer review only - http://bmjopen.bmj.com/site/about/guidelines.xhtml

compared to core areas; and (3) small areas with better socioeconomic characteristics have a higher life expectancy at birth.

Methods

Study setting

We used data compiled by the SALURBAL project (Urban Health in Latin America; 'Salud Urbana en América Latina') for the city of Córdoba during the years 2015-2018, which has compiled and harmonized data on health, social, and built environment indicators for a large number of cities in different Latin American countries.[20] The city of Córdoba (Argentina), the capital and main city of Córdoba province, is a highly urbanized area of 576 km^2 and around 1.3 million residents. We defined small areas as the 99 *fracciones censales* (spatially delimited units used for census data collection). These small areas had a median population (5th-95th percentile) of 13,370 (5,837-22,031) individuals in 2010 (median annual average for the 2015-2018 period: 14,389 (6,305-23,567)).

Data Sources

We used data from (1) death registries georeferenced to small areas during the years 2015-2018, (2) projected population estimates by age, sex, and municipality for 2015-2018, and (3) population and socioeconomic characteristics by small area from the 2010 national population census of Argentina.[21]

Mortality Data

Decedents' residential addresses were extracted from death certificates provided by the Civil Registry of the City of Córdoba, and were georeferenced to latitude and longitude coordinates using the ESRI World Geocoding Service by the SALURBAL team. We used all mortality records for the city of Córdoba from 2015 to 2018 (n=42,115) that included residential addresses of the deceased. We georeferenced these records to one of 99 small areas, and excluded records georeferenced to areas outside of the administrative boundaries of Córdoba (n=1,084) and to non-residential points of interest (n=133). Of the remaining 40,898 georeferenced death records, n=4,075 (~10%) had low georeferencing accuracy (i.e., matched to street name but not building number) but could still be assigned to a small area using the center point of the street or area they were georeferenced to, and were included in the study.

Population Data

First, we obtained population denominators by sex, single years of age (0 to 110+ years), and small areas from 2010 Argentina census and calculated the proportion of people in the city of Córdoba by age, sex, and small area. Second, we obtained projected population estimates provided by the National Institute of Statistics and Census of Argentina for 2015-2018 by 5-year age groups (0 to 85+ years) and sex for the whole municipality of Córdoba and graduated this data into single ages using a penalized composite link model (PCLM).[22] We then obtained small-area population by single age and sex for 2015-2018 by applying the 2010 census proportions to the graduated municipality-level population (2015-2018).

Socioeconomic characteristics of small areas

For peer review only - http://bmjopen.bmj.com/site/about/guidelines.xhtml

We used the following variables to proxy socioeconomic characteristics of small areas: % of the population aged 15 to 17 years attending school, % of the population aged 25 years or above with completed secondary education or above, % of households with piped water access inside the dwelling, % households with overcrowding (defined as more than three people per room), and unemployment rate among individuals aged 15 years and above. Area-level education has been associated with variations in intra-urban mortality in Argentina,[4,16] and water access, sanitation, and less overcrowding[18] have been also associated with higher life expectancy in Latin American cities. In addition, and in order to capture broader features of the social environment, we created a composite z-score index combining the variables listed above. Although a multidimensional index only captures some aspects of the urban infrastructure and resources,[18] it is likely a better proxy of the social environment than a single indicator. Combined indices including education indicators have been frequently used in health disparities research.[23,24] Before creating the composite z-score index, we standardized each of these variables (unemployment and overcrowding were reversed by multiplying with -1). The average of these standardized scores was defined as the composite z-score representing the socioeconomic characteristics of the small areas.

Statistical analysis

To estimate life expectancy, we need an estimate of age-, sex-, and small-area-specific mortality rates. We estimated these through a Bayesian adaptation of the tool for projecting age patterns using linear splines method (TOPALS), incorporating spatial smoothing between small areas.[25] TOPALS regression requires the use of a standard mortality schedule. The core idea is that the schedule of log mortality rates to be estimated is the sum of the standard schedule and a linear spline function. Following the original TOPALS method,[25] we used the city-level Córdoba mortality schedule as the standard schedule. Other research has shown that the choice of the standard mortality schedule does not fundamentally alter results.[26] Because the log mortality rates of the standard mortality schedule were noisy (i.e., contained unexplained variance) (see Supplementary material, Figure S1), we fit a LOESS regression, and the resulting smoothed rates were used as the standard mortality schedule. The models were run in WinBUGS[27] by using the R package R2WinBUGS[28] for 100,000 iterations and the first 80,000 samples were discarded as *burn-ins*. The remaining samples were thinned by a factor of 10 to reduce the autocorrelation of the samples.[18] We fit the models for women and men separately. Eventually, we retained 2,000 sets of age-, sex-, and small-area- specific mortality rates from the posterior distribution. See the Supplementary material for further details on how mortality rates were modeled.

To calculate life expectancy at birth, we inputted age- and sex-specific mortality rates into single-age life tables using the DemoTools package in R.[29] Life expectancy at birth is a simple indicator to present differences in mortality across and within populations,[30] and is defined as the number of years someone born today is expected to live if current age-specific mortality patterns hold constant in the future. These life tables were calculated for each of the 2,000 sets of mortality rates resulting in 2,000 sex- and small-area- specific life expectancy at birth estimates. For descriptive purposes, we report a point estimate (median) and 95% credible intervals (2.5th and 97.5th percentiles). We also extracted life expectancy at ages 20, 40, and 60 years from life tables to explore what ages could drive differences in life expectancy at birth.

To estimate the amount of variability in life expectancy at birth for each sex (hypothesis 1), we calculated the difference between 90th and the 10th percentile of the distribution of life expectancy

For peer review only - http://bmjopen.bmj.com/site/about/guidelines.xhtml

at birth (P90-P10 gap) across small areas. The P90-P10 gap represents the variability in life expectancy across the city. To analyze the geographical patterning of life expectancy at birth (hypothesis 2), we presented choropleth maps of life expectancy at birth for men and women in small areas using ArcGIS. Finally, to examine the association between life expectancy at birth and socioeconomic characteristics of small areas (hypothesis 3), we fit univariate linear regressions of life expectancy on each predictor variable converted into deciles and scored on a continuous scale between 0 to 1. Specifically, for each socioeconomic variable, we assigned the value of 0 if it corresponds to the first decile of its distribution across all small areas. The second decile got the value of 1/9, the third decide got the value of 2/9, and so on. To acknowledge uncertainty around the estimates of life expectancy, these linear regressions were repeated 2,000 times, one per life expectancy estimate. We used Rubin's formula to pool coefficients to obtain a single regression coefficient and associated standard errors. Each coefficient represents the mean difference in life expectancy in areas with the highest socioeconomic variable (i.e., those in the tenth decile) versus the areas with the lowest value of the socioeconomic variable (those in the first decile), and is presented as the Slope Index of Inequality (SII). Linear regression models were run using PROC REG and PROC MIANALYZE in SAS software.

Patient and Public Involvement

SALURBAL has launched a series of Knowledge-to-Policy Fora to present preliminary results and engage urban health policy actors from across Latin America in dialogue on urban health research and policy priorities in the region. For more information see https://drexel.edu/lac/events-workshops/knowledge-policy-forum/. The project also holds and participates in additional periodic workshops to engage stakeholders in activities designed to disseminate research findings and engage stakeholders in systems thinking around urban policies.

Results

A median of 222 and 199 deaths occurred during the study period per small area among women and men, respectively (Table 1). A median of 85.3% of adolescents between 15-17 years attended school, 58.4% of people had minimum high school education, and 7.0% were unemployed (Table 1). Moreover, 97.6% of dwellings had inside water connections and 2.1% of households experienced overcrowding. Median life expectancy at birth was 80.3 years for women with variability in life expectancy (P90-P10 gap) of 3.2 years (P90=81.5 years and P10=78.3 years) (Table 1). Among men, there was lower median life expectancy at birth (75.1 years), with a gap (P90-P10) of 4.6 years between small areas (P90=77.1 years, and P10=72.5 years). For the full distribution of life expectancy at birth and uncertainty around the estimates see Figure S2 (Supplementary material).

Table 1. Number of deaths, life expectancy among women and men, and selected sociodemographic characteristics for small areas (n=99). Córdoba, 2015-2018.

	Median (10th–90th percentile)	
	Women	Men
Number of deaths	222 (90, 340)	199 (81, 309)
Estimated population, annual average [a]	7,326 (3,818, 11,157)	6,874 (3,214, 10,534)
Life expectancy at birth, years [a]	80.3 (78.3, 81.5)	75.1 (72.5, 77.1)
Life expectancy at age 20, years [a]	61.0 (59.1, 62.2)	56.2 (53.6, 57.9)

For peer review only - http://bmjopen.bmj.com/site/about/guidelines.xhtml

Life expectancy at age 40, years [a]	41.5 (39.8, 42.6)	36.9 (34.7, 38.6)
Life expectancy at age 60, years [a]	23.3 (21.6, 24.3)	19.2 (17.2, 21.0)
Population aged 15 or younger, % [a]	22.0 (8.7, 30.3)	25.1 (10.6, 32.2)
Population aged 65 or older, % [a]	13.3 (7.1, 19.5)	8.9 (5.3, 12.5)
Households with water inside dwelling, % [b]	97.6 (89.8, 99.4)	
Households with overcrowding, % [b]	2.1 (0.4, 7.1)	
School attendance among 15-17 years old, % [b]	85.3 (76.2, 91.9)	
Adults with high school education or above, % [b]	58.4 (30.1, 88.7)	
Unemployment, % [b]	7.0 (4.6, 9.7)	

Footnote: [a] Data from 2015-2018; [b] Data from 2010 census; Overcrowding: proportion of households with more than three people per room.

There were higher life expectancies in the core and northwestern parts of the city for both men and women, along with the southwestern part of the city for men (Figure 1). The distribution of life expectancy at ages 20, 40, and 60 years showed similar spatial patterns to those for life expectancy at birth in women and men (see Figure S3, Supplementary material).

[Figure 1]

Overall, there were positive associations between life expectancy at birth and better socioeconomic characteristics of small areas (Figure 2, Table 2), with associations being slightly stronger among men. Living in small areas with the highest compared to the lowest decile of high school education and school attendance was associated with longer life expectancy (SII=3.03 and 3.52 years, respectively in men and 2.58 and 2.56 years, respectively in women) (Table 2). Similarly, higher proportions of households with water inside the dwelling was associated with longer life expectancy by 2.97 years in men, and 2.31 years in women, while lower overcrowding and % unemployment was associated with shorter life expectancy at birth by 3.03 and 2.73 years, respectively, and 2.57 and 2.34 years for women (Table 2). The composite z-score was also strongly associated with life expectancy, as higher values (indicating better living conditions) were associated with 3.27 and 2.69 years longer life expectancy in men and women, respectively.

Table 2. Slope Index of Inequality (SII) in life expectancy (years) associated with small area characteristics among women and men, Córdoba city, 2015-2018.

Variable/model	Slope Index of Inequality (95% Confidence Intervals)	
	Women	Men
% with High school education or above	2.58 (1.63, 3.52)	3.03 (1.77, 4.30)
% School attendance among adolescents (15-17 years)	2.56 (1.65, 3.47)	3.52 (2.35, 4.69)
% households with water inside dwelling	2.31 (1.36, 3.26)	2.97 (1.72, 4.22)
% households with overcrowding	- 2.57 (-3.51, -1.63)	- 3.03 (-4.29, -1.77)
% Unemployment	- 2.34 (-3.27, -1.41)	- 2.73 (-3.98, -1.49)
Composite z-score	2.69 (1.78, 3.60)	3.27 (2.05, 4.48)

For peer review only - http://bmjopen.bmj.com/site/about/guidelines.xhtml

Footnote: the models were run in a univariate fashion, one variable at a time. Small-area characteristics were transformed into deciles. The Slope Index of Inequality (SII) represents the mean difference in life expectancy in areas with the highest predictor variable (i.e., those in the tenth decile, having value=1) versus the areas with the lowest value of the predictor variable (those in the first decile, having value=0) as estimated from a linear regression including all deciles. Socioeconomic data for small areas came from the 2010 census; Overcrowding: proportion of households with more than three people per room.

[Figure 2]

Discussion

Our study evaluated the variability in life expectancy at birth, described the spatial patterning of life expectancy, and examined the association between life expectancy and socioeconomic characteristics of small areas in the city of Córdoba (Argentina) during the 2015-2018 period. We found a higher life expectancy for women (5.2 years higher than for men), that life expectancy varied within the city of Córdoba, with a P90-P10 gap of 4.6 years for men and 3.2 years for women, and that life expectancy was higher in the central and northwestern parts of the city, along with the southwestern part in the case of men. Lastly, we found that a series of small-area socioeconomic characteristics were highly predictive of life expectancy.

Analyses of spatial variability provide critical information for understanding place effects on health.[31] We found considerable variability (P90-P10 gap) in life expectancy at birth across small areas of Córdoba, with this gap being larger in men (4.6 years) than in women (3.2 years). In another study, we found that variability in rates of non-communicable disease risk factors was larger across neighborhoods within cities than across cities,[32] further highlighting the importance of within-city inequities. A recent study on the variations in life expectancy across states, counties, and census tracts in the US showed that more than three-fourths of the total variation in life expectancy was attributable to census tract units, suggesting that large heterogeneities in longevity occur at a local level.[9]

Some prior work has demonstrated within-city heterogeneities in life expectancy in lower- and middle-income countries. A prior SALURBAL paper based on six large cities in the region found that gaps in life expectancy across subcity units ranged from a high of 15.0 years in men from Panama City (Panamá) to a low of 3.0 years in women from San José (Costa Rica).[16] However, this analysis focused on only the largest cities and also investigated subcity areas of varying size but all larger than the ones we investigated in Córdoba. Very few studies examined variation across smaller areas and most of these were in large cities. A study in Rio de Janeiro[33] showed that life expectancy at birth of men living in the richest parts of the city was 12.8 years longer than that of men living in deprived areas. In Argentina, one prior study focused on the city of Buenos Aires demonstrated large variations in mortality rates across censal fractions but life expectancy differences were not explored.[4]

Comparison across studies in the size of within-city differences is rendered complex by the use of geographic units of different sizes and the way in which the city is defined. For example, some articles[16] have used larger sub city geographic units (which could perhaps hide heterogeneity) but have also employed a broader definition of cities, encompassing peripheral areas around the core city that are often poorer (possibly leading to the larger estimates of within-city differences). The metrics used to characterize within-urban differences also vary. We compared the 90th to the 10th percentile to avoid excessive influence of extreme values but others compare maximum and

For peer review only - http://bmjopen.bmj.com/site/about/guidelines.xhtml

minimum.[7,11] On a more substantive level, the degree of residential segregation may also impact the magnitude of heterogeneity in life expectancy across areas within a city.[11] Further work is needed to contrast and understand the drivers of differences across cities in the size of small-area differences in life expectancy.

In addition to documenting large heterogeneity across areas, we also found that life expectancy at birth was geographically patterned, with higher life expectancy in the core and northwestern parts of the city, for both men and women, along with the southwestern part of the city for men. This suggested that variability in life expectancy was not random in Córdoba. Indeed, the city of Córdoba has an important socioeconomic residential segregation[34] that matched the spatial pattern of life expectancy: while the north-east-south periphery of the city predominately includes residential areas with poverty segregation, the central core, and the northwest corridor are characterized by a greater concentration of wealth. There has been growing spatial concentration and social isolation of households with a lower socioeconomic position as a consequence of public housing policies implemented in recent decades in the city of Córdoba. Particularly, relocations from informal settlements to large housing complexes built in the poor and semi-rural periphery of the city have been implemented by the provincial government between 2003 and 2007.[34,35] The spatial pattern in Córdoba, which exhibits the highest life expectancy in the core and the northwest, was more clearly defined for women, while we also found higher life expectancy in the southern periphery among men. Previous research showed a similar core-periphery divide[16] and a north-to-south pattern in Buenos Aires, which can be explained to some extent by the spatial patterning of socioeconomic deprivation. Our findings emphasize the need to address spatial variation in health within cities in further studies in the local context.

We also explored factors that might explain the heterogeneity in life expectancy across small areas. We found positive associations between improved socioeconomic characteristics of small areas and life expectancy at birth in both men and women, suggesting that the described heterogeneity in life expectancy might be driven by the socioeconomic features of small areas. All factors including educational attainment, school attendance, water access, overcrowding, unemployment, and the composite index were significantly associated with life expectancy at birth in the expected direction. These associations were stronger among men, a finding that could be driven by certain causes of death and that deserves further exploration. Our findings are also consistent with other research in Argentina describing a negative association between an area-level deprivation index and life expectancy at birth in 32 urban agglomerations,[24] and a positive association between mortality rate and unmeet basic needs in Departamentos of the "Pampeana" region.[36] Furthermore, these results are consistent with factors associated with within-city inequalities in life expectancy in six large Latin American cities.[16]

A key strength of our analyses is that we employed a novel Bayesian approach that draws strength and smooths estimates from surrounding small areas and from the overall structure of the mortality schedule. This allowed us to derive valid and reliable estimates for small areas even in the presence of sparse data.[37] We also characterized associations with socioeconomic variables using the SII, a measure that uses the full distribution to derive a global measure of inequities. By using the SII based on deciles we avoided the undue influence of extreme values of socioeconomic factors. The use of the SII defined in this way will also allow easier comparisons across cities. While some Latin American countries have vital registration records with incomplete registration, this is less of a concern in Argentina.[18,38,39] Another strength is that we examined these associations using univariate

For peer review only - http://bmjopen.bmj.com/site/about/guidelines.xhtml

models that acknowledge uncertainty around the estimates of life expectancy by applying multiple imputation type techniques.

Among the limitations of our study was the relatively high proportion of records that had low georeferencing accuracy, i.e., that were georeferenced to the street level. It was likely that these records were not randomly distributed. In fact, low georeferencing accuracy seemed to impact mostly small areas in the periphery. Given the challenges of sparse data, we also needed to apply spatial and age smoothing to address the small-area estimation of mortality rates. This smoothing could have led to an underestimation of inequalities, as reported in previous research.[16] Because annual small-area population by age and sex were not available, we had to estimate these by combining official municipality-level projected population estimates from 2015-2018 with the most recent census in 2010. If the population age or sex structure changed over time differentially by area, our estimates may be biased. Relatedly, social characteristics of small areas were retrieved from the same 2010 census and do not align with the years for which death records were obtained (2015-2018). We, therefore, assumed that social characteristics were relatively stable across the years examined. While ideally this analysis would have used census data from other years to better assess small-area changes over time, we could only rely on the 2010 census because small-area data from the 2001 Argentina census were not available, and the 2020 census was postponed to 2022 due to the COVID-19 pandemic, and data are not available yet. We explored life expectancy as it is a commonly used metric but other metrics such as age at death may also be of value to examine.

Conclusion

In summary, we find that spatial variations in life expectancy across small areas within cities linked to small-area social and economic conditions extend beyond high-income countries and beyond the very large metropolitan areas in which they have been predominantly studied. These within-cities heterogeneity that are likely present or emerging in many small- and middle-sized cities all over the world are of major relevance for urban policies to promote health and achieve health equity in low- and middle-income countries.

Authors' contributions

SRL, NT, UB, KM and AVDR, conceived the study. BA and HQ did the statistical analyses. SRL, NT, and BA drafted the first version of the manuscript. SRL, NT, KM, ADQ, GEA and AVDR participated in or supported data collection. All authors participated in the interpretation of results and approved the final version of the manuscript.

Funding

The Salud Urbana en América Latina (SALURBAL)/ Urban Health in Latin America project is funded by the Wellcome Trust [205177/Z/16/Z]. NT was awarded by the European Union's Horizon 2020 research and innovation programme with the Marie Sklodowska-Curie grant agreement N◦ 89102. UB was supported by the Office of the Director of the National Institutes of Health under award number DP5OD26429.

Competing interest statement

For peer review only - http://bmjopen.bmj.com/site/about/guidelines.xhtml

We declare no competing interests.

Role of funding source

The funder of the study had no role in study design, data collection, data analysis, data interpretation, or writing of this article.

Ethics approval statement

The SALURBAL study protocol was approved by the Drexel University Institutional Review Board with ID #1612005035 and by appropriate site-specific IRBs.

Data sharing statement

Interested parties could submit a proposal to SALURBAL to use the vital registration data at the small-area level and sign a data use agreement if approved. The SALURBAL project welcomes queries from anyone interested in learning more about its dataset and potential access to data. To learn more about SALURBAL's dataset, visit https://drexel.edu/lac/ or contact the project at salurbal@drexel.edu.

Acknowledgements

The authors acknowledge the contribution of all SALURBAL project team members. For more information on SALURBAL and to see a full list of investigators see https://drexel.edu/lac/salurbal/team/. We would like to thank Steve Melly for his help in creating the maps for this study. We thank Eugenia Peisino and Alicia Díaz for their help in accessing to the mortality data.

References

1 de Snyder VNS, Friel S, Fotso JC, *et al.* Social conditions and urban health inequities: Realities, challenges and opportunities to transform the urban landscape through research and action. *J Urban Health* 2011;**88**:1183–93. doi:10.1007/s11524-011-9609-y

2 Borrell C, Pons-Vigués M, Morrison J, *et al.* Factors and processes influencing health inequalities in urban areas. *J Epidemiol Community Health* 2013;**67**:389–91. doi:10.1136/jech-2012-202014

3 Diez Roux AV, Mair C. Neighborhoods and health. *Ann N Y Acad Sci* 2010;**1186**:125–45. doi:10.1111/j.1749-6632.2009.05333.x

4 Diez Roux AV, Green Franklin T, Alazraqui M, *et al.* Intraurban Variations in Adult Mortality in a Large Latin American City. *J Urban Health* 2007;**84**(3):319-333. doi: 10.1007/s11524-007-9159-5

5 Emerging and Sustainable Cities Program | IDB. https://www.iadb.org/en/urban-development-and-housing/emerging-and-sustainable-cities-program (accessed 30 Jul 2022).

For peer review only - http://bmjopen.bmj.com/site/about/guidelines.xhtml

6 UN Habitat. *State of the world's cities 2012/2013: Prosperity of cities*. Taylor and Francis 2013. doi:10.4324/9780203756171

7 Moser A, Panczak R, Zwahlen M, *et al.* What does your neighbourhood say about you? A study of life expectancy in 1.3 million Swiss neighbourhoods. *J Epidemiol Community Health* 2014;**68**:1125–32. doi:10.1136/JECH-2014-204352

8 Dwyer-Lindgren L, Stubbs RW, Bertozzi-Villa A, *et al.* Variation in life expectancy and mortality by cause among neighbourhoods in King County, WA, USA, 1990–2014: a census tract-level analysis for the Global Burden of Disease Study 2015. *Lancet Public Health* 2017;**2**:e400–10. doi:10.1016/S2468-2667(17)30165-2

9 Boing AF, Boing AC, Cordes J, *et al.* Quantifying and explaining variation in life expectancy at census tract, county, and state levels in the United States. *Proc Natl Acad Sci* 2020;**117**:17688–94. doi:10.1073/PNAS.2003719117

10 Tobias MI, Cheung J. Monitoring health inequalities: life expectancy and small area deprivation in New Zealand. *Popul Health Metr* 2003;**1**:1–11. doi:10.1186/1478-7954-1-2

11 Melix BL, Uejio CK, Kintziger KW, *et al.* Florida neighborhood analysis of social determinants and their relationship to life expectancy. *BMC Public Health* 2020;**20**:1–12. doi:10.1186/S12889-020-08754-X/FIGURES/1

12 Sudharsanan N, Zhang Y, Payne CF, *et al.* Education and adult mortality in middle-income countries: Surprising gradients in six nationally-representative longitudinal surveys. *SSM Popul Health* 2020;**12**. doi:10.1016/J.SSMPH.2020.100649

13 Pou S, Tumas N, Sánchez Soria D, *et al.* Large-scale societal factors and noncommunicable diseases: Urbanization, poverty and mortality spatial patterns in Argentina. *Appl Geogr* 2017;**86**:32–40. doi:http://dx.doi.org/10.1016/j.apgeog.2017.06.022

14 De Maio FG. Ecological analysis of the health effects of income inequality in Argentina. *Public Health* 2008;**122**:487–96. doi:http://dx.doi.org/10.1016/j.puhe.2007.09.006

15 Macchia A, Mariani J, Nul D, *et al.* Social inequity, place of residence and all-cause premature death in Argentina. *Rev Arg Cardiol* 2016;**84**:108–13. doi:10.7775/RAC.V84.I2.8267

16 Bilal U, Alazraqui M, Caiaffa WT, *et al.* Inequalities in life expectancy in six large Latin American cities from the SALURBAL study: an ecological analysis. *Lancet Planet Health* 2019;**3**:e503–10. doi:https://doi.org/10.1016/S2542-5196(19)30235-9

17 James C. Featured Graphic. Lives on the Line: Mapping Life Expectancy along the London Tube Network. *Environ Plan A* 2012;**44**:1525–8. doi:10.1068/A45341

18 Bilal U, Hessel P, Perez-Ferrer C, *et al.* Life expectancy and mortality in 363 cities of Latin America. *Nat Med* 2021;**27**:463–70. doi:https://doi.org/10.1038/s41591-020-01214-4

19 Kii M. Projecting future populations of urban agglomerations around the world and through the 21st century. *npj Urban Sustain* 2021;**1**:1–12. doi:10.1038/s42949-020-00007-5

For peer review only - http://bmjopen.bmj.com/site/about/guidelines.xhtml

20 Quistberg DA, Diez Roux AV, Bilal U, *et al.* Building a Data Platform for Cross-Country Urban Health Studies: the SALURBAL Study. *J Urban Health* 2019;**96**:311–37. doi:10.1007/s11524-018-00326-0

21 INDEC. Censo Nacional de Población, Hogares y Viviendas 2010 [National Census of Population, Households and housing 2010]. 2010.

22 Rizzi S, Gampe J, Eilers PHC. Efficient Estimation of Smooth Distributions from Coarsely Grouped Data. *Am J Epidemiol* 2015;**182**:138–47. doi:10.1093/aje/kwv020

23 Durán RJ, Condorí MÁ. Deprivation Index for Small Areas Based on Census Data in Argentina. *Soc Indic Res* 2019;**141**:331–63. doi:10.1007/s11205-017-1827-6

24 Ivaldi E, Parra Saiani P, Primosich JJ, *et al.* Health and Deprivation: A New Approach Applied to 32 Argentinian Urban Areas. *Soc Indic Res* 2020;**151**:155–79. doi:10.1007/s11205-020-02369-w

25 Gonzaga MR, Schmertmann CP. Estimating age- and sex-specific mortality rates for small areas with topals regression: An application to Brazil in 2010. *Rev Bras Estud Popul* 2016;**33**:629–52. doi:10.20947/S0102-30982016c0009

26 Schmertmann CP, Gonzaga MR. Bayesian Estimation of Age-Specific Mortality and Life Expectancy for Small Areas With Defective Vital Records. *Demography* 2018;**55**:1363–88. doi:10.1007/S13524-018-0695-2

27 Lunn D, Thomas A, Best N, *et al.* WinBUGS — a Bayesian modelling framework: concepts, structure, and extensibility. *Stat Comput* 2000;**10**:325–37.

28 Sturtz S, Ligges U, Gelman A. R2WinBUGS: A Package for Running WinBUGS from R. *J Stat Softw* 2005;**12**:1–16.

29 Riffe T, Aburto JM, Alexander M, *et al.* DemoTools: An R package of tools for aggregate demographic analysis. 2019.https://github.com/timriffe/DemoTools/

30 Modig K, Rau R, Ahlbom A. Life expectancy: what does it measure? *BMJ Open* 2020;**10**:e035932. doi:10.1136/bmjopen-2019-035932

31 Merlo J, Viciana-Fernandez F, Ramiro-Farinas D, *et al.* Bringing the individual back to small-area variation studies: A multilevel analysis of all-cause mortality in Andalusia, Spain. *Soc Sci Med* 2012;**75**:1477–87.

32 Tumas N, Rodríguez López S, Bilal U, *et al.* Urban social determinants of non-communicable diseases risk factors in Argentina. *Health Place* 2021:102611. https://doi.org/10.1016/j.healthplace.2021.102611

33 Szwarcwald CL, Mota JC da, Damacena GN, *et al.* Health Inequalities in Rio de Janeiro, Brazil: Lower Healthy Life Expectancy in Socioeconomically Disadvantaged Areas. *Am J Public Health* 2011;**101**:517–23. doi:10.2105/AJPH.2010.195453

34 Molinatti F. Segregación residencial socioeconómica en la ciudad de Córdoba (Argentina): Tendencias y patrones espaciales. *Rev INVI* 2013;**28**:61–94.

For peer review only - http://bmjopen.bmj.com/site/about/guidelines.xhtml

35 Marengo C. Estrategias habitacionales en los bordes urbanos: puntos de partida para analizar la expansion urbana. *Proyección* 2003; 2:9p. https://bdigital.uncu.edu.ar/3112

36 Sacco N, Williams I, L. Queiroz B. Estimaciones de esperanza de vida al nacer en áreas menores de la región pampeana. *Rev Bras Estud Popul* 2021;**38**:1–41. doi:10.20947/s0102-3098a0160

37 Jonker MF, van Lenthe FJ, Congdon PD, *et al.* Comparison of Bayesian Random-Effects and Traditional Life Expectancy Estimations in Small-Area Applications. *Am J Epidemiol* 2012;**176**:929–37. doi:10.1093/AJE/KWS152

38 Peralta A, Benach J, Borrell C, *et al.* Evaluation of the mortality registry in Ecuador (2001–2013) – social and geographical inequalities in completeness and quality. *Popul Health Metr* 2019;**17**:1–12. doi:10.1186/S12963-019-0183-Y

39 Adair T, Lopez AD. Estimating the completeness of death registration: An empirical method. *PLoS ONE* 2018;**13**:e0197047. doi:10.1371/JOURNAL.PONE.0197047

For peer review only

Figure legends

Figure 1. Spatial distribution of life expectancy at birth in women and men in the city of Córdoba, 2015-2018.

Footnote: All small areas (n=99) of the city are indicated. To facilitate within-sex comparisons, each map uses its own legend.

Figure 2. Association between life expectancy at birth and socioeconomic characteristics of small areas in women and men. Córdoba, 2015-2018.

Footnote: Blue line refers to the linear fit.

For peer review only

For peer review only - http://bmjopen.bmj.com/site/about/guidelines.xhtml

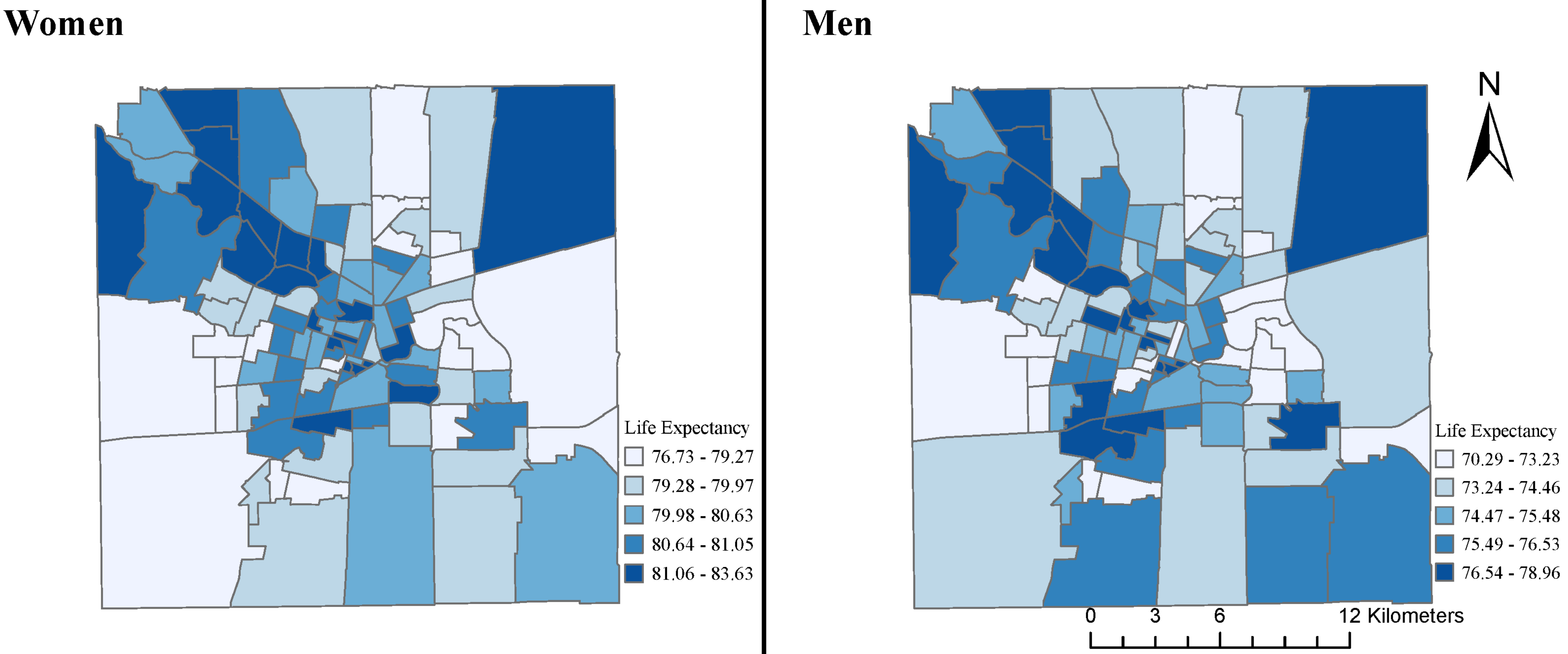
Women
Men
N
Life Expectancy
76.73 - 79.27
79.28 - 79.97
79.98 - 80.63
80.64 - 81.05
81.06 - 83.63
Life Expectancy
70.29 - 73.23
73.24 - 74.46
74.47 - 75.48
75.49 - 76.53
76.54 - 78.96
0
3
6
12 Kilometers

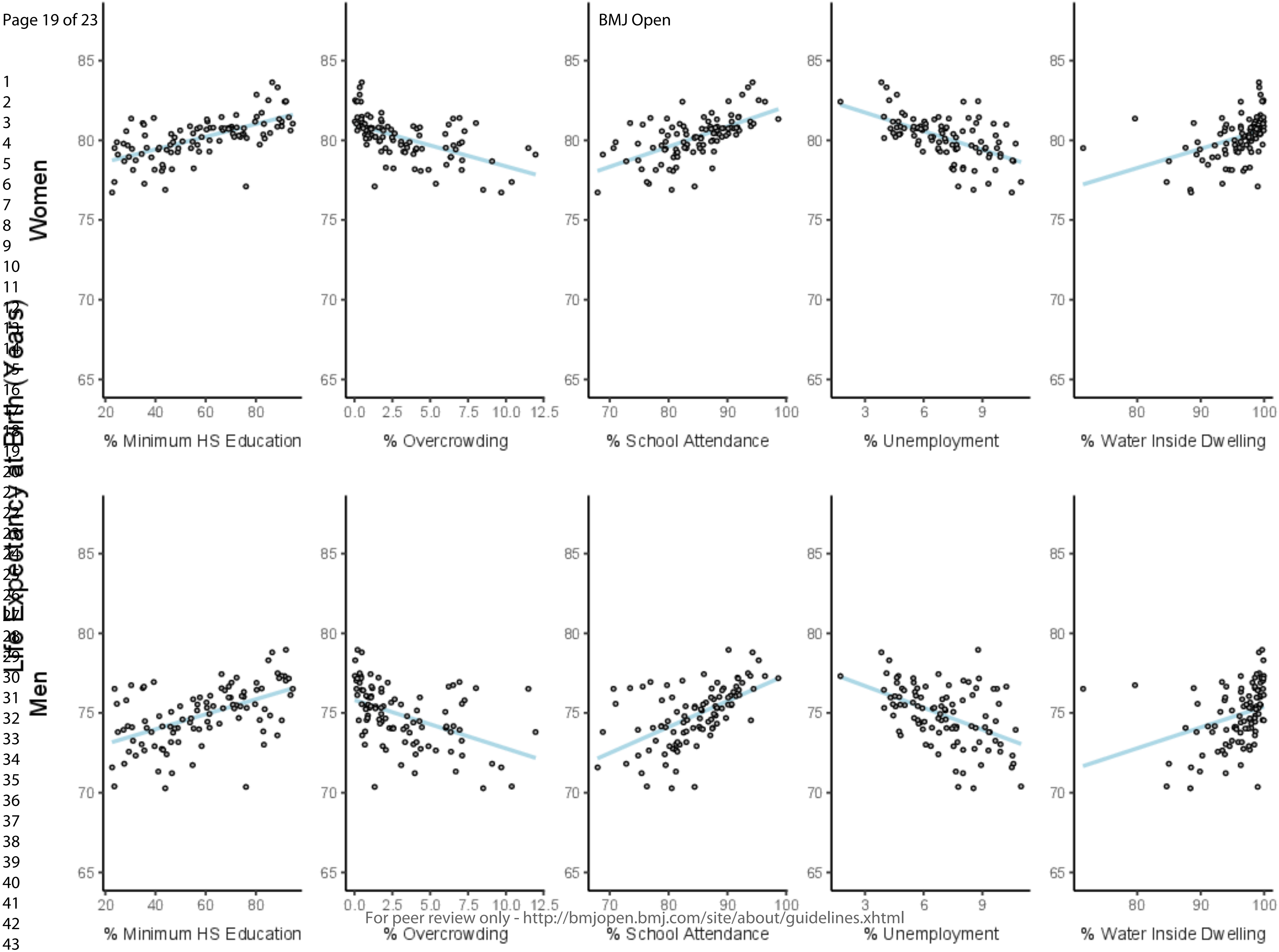
Life Expectancy at Birth (Years)
Women
Men
85
80
75
70
65
20 40 60 80
% Minimum HS Education
0.0 2.5 5.0 7.5 10.0 12.5
% Overcrowding
70 80 90 100
% School Attendance
3 6 9
% Unemployment
80 90 100
% Water Inside Dwelling

Supplementary material

Modeling the mortality rate

We employ the Bayesian adaptation of the tool for projecting age patterns using linear splines method (TOPALS) which incorporates spatial smoothing in small areas.[19]

For age a $\{a = 0, 1, 2, \ldots, 85\}$ and small area i $\{i = 1, 2, \ldots, 99\}$ with corresponding population n_{ia}, we assume that the number of deaths comes from a Poisson distribution with underlying rate λ_{ia}.

$$y_{ia} | \lambda_{ia} \sim Pois\ (n_{ia}\, \lambda_{ia})$$

We further assume that the vector of log mortality rates in small area i is,

$$log(\boldsymbol{\lambda}_{i.}) = log(\boldsymbol{\lambda}^{*}) + \boldsymbol{B}\, \boldsymbol{\beta}_{k.}\,,$$

Where $\boldsymbol{\lambda}_{i.}$ is the vector with elements λ_{ia} representing age-specific mortality rates in small areas, $\boldsymbol{\lambda}^{*}$ is the vector of standard mortality schedule (i.e., the smoothed city-level rates), $\boldsymbol{B}$ is a matrix of constants of size 86×7 in which each column is a linear B-spline basis function, and $\boldsymbol{\beta}_{k.}$ is a vector of parameters with elements β_{ik} representing offsets to the standard schedule.

We define knots at ages $t_0, \ldots, t_6$ = (0, 1, 10, 20, 40, 70, 85). For ages a in {0, 1, 2, ..., 85} and columns k in {1,...,7} the basis functions in $\boldsymbol{B}$ are:

$$\frac{a - t_{k-1}}{t_k - t_{k-1}}\ if\ t_{k-1} \leq\ a \leq t_k;$$

$$\frac{t_{k+1} - a}{t_{k+1} - t_k}\ if\ t_k \leq\ a \leq t_{k+1};$$

$$0\ \ otherwise.$$

We further decompose the β_{ik} into the intercepts at each knot, β_{0k}; the spatial random effects, z_{ik}; and unstructured random effects, ϕ_{ik} that vary by knot age and area.

$$\beta_{ik} = \beta_{0k} + z_{ik} + \phi_{ik}\,.$$

We assign the unstructured, non-spatial random effect (ϕ_{ik}) an exchangeable zero-mean normal prior with the knot-specific variance $\sigma^2_{ns;\,k}$. The variance parameter in turn receives the uninformative inverse gamma hyper-prior with the shape and rate parameter of 1 and 0.01, respectively.

That is,

$$\phi_{ik} \sim Normal\ (0, \sigma^2_{ns;\,k})$$
$$\sigma^2_{ns;\,k} \sim Inverse\ Gamma\ (1, 0 \cdot 01)$$

For the intercept β_{0k}, we assign a vague normal prior with mean 0 and variance of 1,000. That is,

$$\beta_{0k} \sim Normal\ (0, 1000)$$

For the spatial random effect z_{ik}, we assign the intrinsic conditional autoregressive (ICAR) prior distribution[45] for each knots, k. We define areas i and j as neighbors if they share one or more common vertex between boundaries, commonly referred to as Queen's contiguity.

For any given knot and for each area i, the conditional expected value of z_{ik} is the mean of its neighboring areas, and the variance of z_{ik} is inversely proportional to the number of neighbors for that area, m_i.

If we drop the subscript for knot here, we can denote the CAR distribution as:

$$z_i | z_{-i}, \boldsymbol{W},\ \sigma_z^2 \sim Normal\ (\bar{z}_i, \frac{\sigma_z^2}{m_i}),$$

where

$$\bar{z}_i = \sum_{j, j \neq i} \frac{w_{i,j}\, z_j}{m_i}$$

and $W = [w_{i,j}]$ is 99 × 99 adjacency matrix with elements $w_{i,j} = 1$ if areas i and j are neighbors and 0 otherwise. We complete the prior specification by assigning a weakly informative inverse gamma prior (1, 0·14) for the variance of CAR random effects.

For peer review only - http://bmjopen.bmj.com/site/about/guidelines.xhtml

Figure S1. Log mortality rates due to all causes for women and men. Córdoba, 2015-2018.

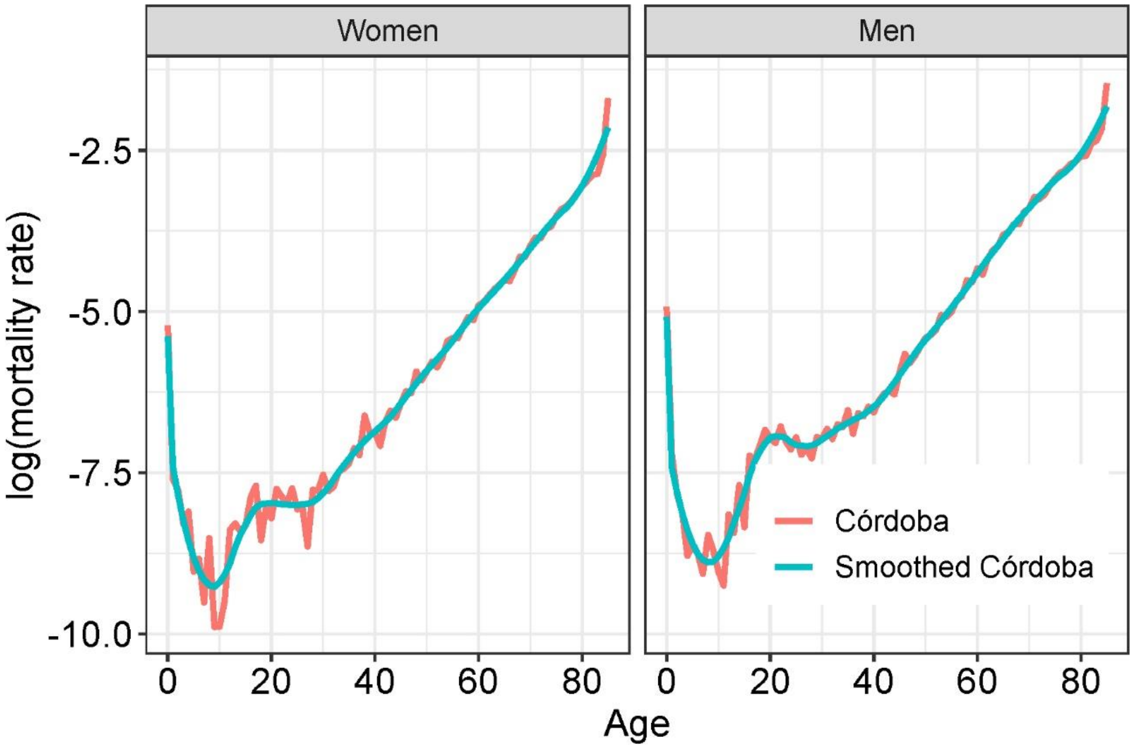

Footnote: because log mortality rates of the standard mortality schedule (labelled Córdoba) were noisy (i.e., contained unexplained variance), we fit a LOESS regression and the resulting LOESS-smoothed curve (labelled Smoothed Córdoba) served as the standard mortality schedule in the analysis.

For peer review only - http://bmjopen.bmj.com/site/about/guidelines.xhtml

Figure S2. Life expectancy at birth (95% Credible Interval) among women and men in small areas. Córdoba, 2015-2018.

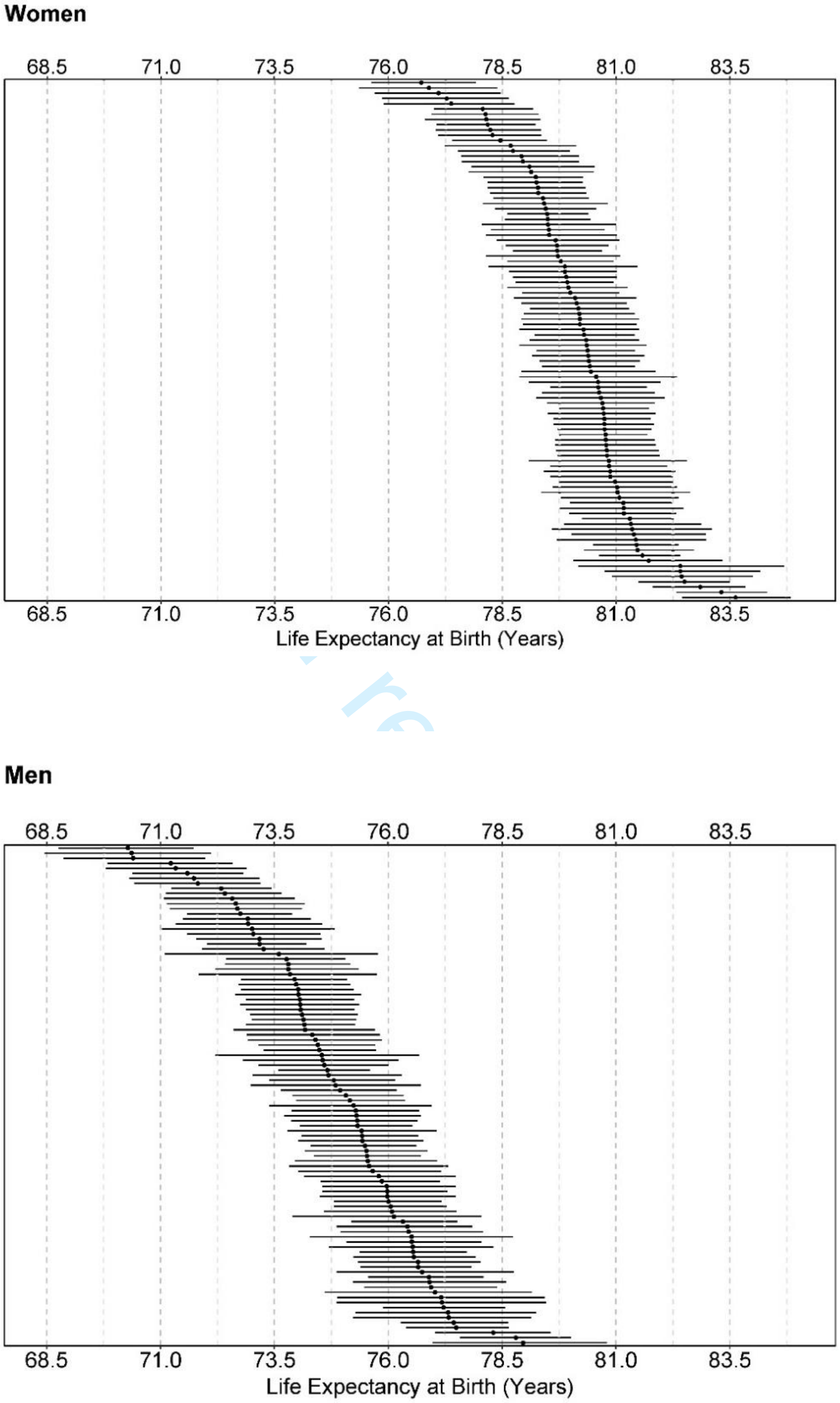

Footnote: small areas are represented in the vertical axis.

For peer review only - http://bmjopen.bmj.com/site/about/guidelines.xhtml

Figure S3. Spatial distribution of life expectancy at birth, 20, 40 and 60 years in women and men in the 99 small areas of city of Córdoba, 2015-2018.

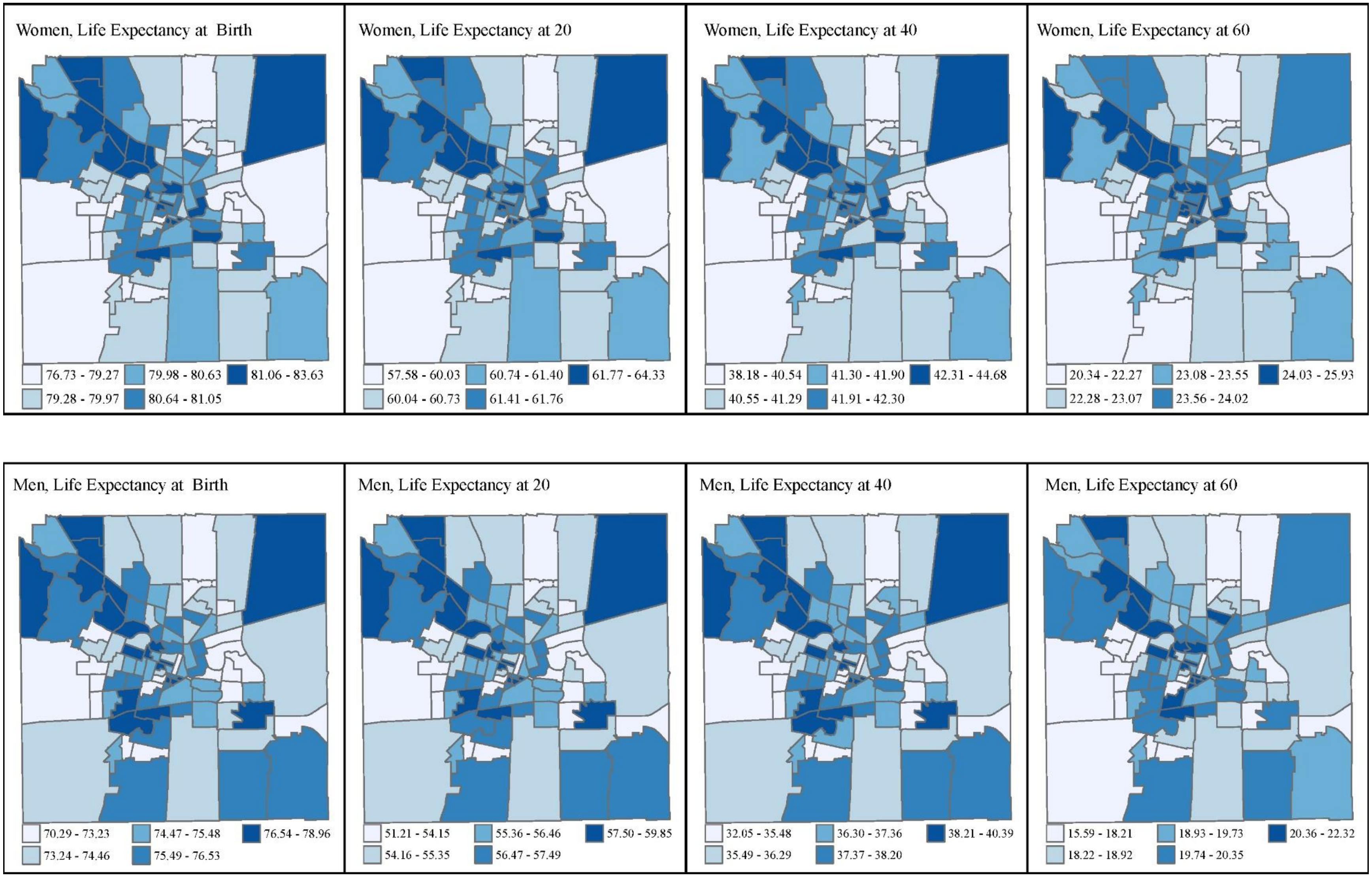

STROBE Statement—Checklist of items that should be included in reports of ***cross-sectional studies***

	Item No	Recommendation	Page No
**Title and abstract**	1	(*a*) Indicate the study's design with a commonly used term in the title or the abstract	1
		(*b*) Provide in the abstract an informative and balanced summary of what was done and what was found	2
**Introduction**			
Background/rationale	2	Explain the scientific background and rationale for the investigation being reported	3
Objectives	3	State specific objectives, including any prespecified hypotheses	3
**Methods**			
Study design	4	Present key elements of study design early in the paper	4
Setting	5	Describe the setting, locations, and relevant dates, including periods of recruitment, exposure, follow-up, and data collection	4
Participants	6	(*a*) Give the eligibility criteria, and the sources and methods of selection of participants	4
Variables	7	Clearly define all outcomes, exposures, predictors, potential confounders, and effect modifiers. Give diagnostic criteria, if applicable	5
Data sources/ measurement	8*	For each variable of interest, give sources of data and details of methods of assessment (measurement). Describe comparability of assessment methods if there is more than one group	4
Bias	9	Describe any efforts to address potential sources of bias	5
Study size	10	Explain how the study size was arrived at	4
Quantitative variables	11	Explain how quantitative variables were handled in the analyses. If applicable, describe which groupings were chosen and why	5
Statistical methods	12	(*a*) Describe all statistical methods, including those used to control for confounding	5-6
		(*b*) Describe any methods used to examine subgroups and interactions	3-4
		(*c*) Explain how missing data were addressed	5
		(*d*) If applicable, describe analytical methods taking account of sampling strategy	
		(*e*) Describe any sensitivity analyses	5
**Results**			
Participants	13*	(a) Report numbers of individuals at each stage of study—eg numbers potentially eligible, examined for eligibility, confirmed eligible, included in the study, completing follow-up, and analysed	4
		(b) Give reasons for non-participation at each stage	5
		(c) Consider use of a flow diagram	
Descriptive data	14*	(a) Give characteristics of study participants (eg demographic, clinical, social) and information on exposures and potential confounders	6
		(b) Indicate number of participants with missing data for each variable of interest	
Outcome data	15*	Report numbers of outcome events or summary measures	6
Main results	16	(*a*) Give unadjusted estimates and, if applicable, confounder-adjusted estimates and their precision (eg, 95% confidence interval). Make clear which confounders were adjusted for and why they were included	6-7

For peer review only

		(*b*) Report category boundaries when continuous variables were categorized	
		(*c*) If relevant, consider translating estimates of relative risk into absolute risk for a meaningful time period	
Other analyses	17	Report other analyses done—eg analyses of subgroups and interactions, and sensitivity analyses	5
**Discussion**			
Key results	18	Summarise key results with reference to study objectives	8
Limitations	19	Discuss limitations of the study, taking into account sources of potential bias or imprecision. Discuss both direction and magnitude of any potential bias	10
Interpretation	20	Give a cautious overall interpretation of results considering objectives, limitations, multiplicity of analyses, results from similar studies, and other relevant evidence	8-9
Generalisability	21	Discuss the generalisability (external validity) of the study results	9
**Other information**			
Funding	22	Give the source of funding and the role of the funders for the present study and, if applicable, for the original study on which the present article is based	10

*Give information separately for exposed and unexposed groups.

Note: An Explanation and Elaboration article discusses each checklist item and gives methodological background and published examples of transparent reporting. The STROBE checklist is best used in conjunction with this article (freely available on the Web sites of PLoS Medicine at http://www.plosmedicine.org/, Annals of Internal Medicine at http://www.annals.org/, and Epidemiology at http://www.epidem.com/). Information on the STROBE Initiative is available at www.strobe-statement.org.

For peer review only
